# Protein Kinase C Family: Structures, Biological Functions, Diseases, and Pharmaceutical Interventions

**DOI:** 10.1002/mco2.70474

**Published:** 2025-11-14

**Authors:** Yongqi Li, Yuhan Jiang, Zhengxi Hu, Shenglan Yang, Longyin Li, Chaohu Xiong, Ya Gao, Weiguang Sun, Yonghui Zhang

**Affiliations:** ^1^ Hubei Key Laboratory of Natural Medicinal Chemistry and Resource Evaluation, School of Pharmacy, Tongji Medical College, Huazhong University of Science and Technology Wuhan China; ^2^ Key Laboratory of Advanced Drug Preparation Technologies, Ministry of Education, China; State Key Laboratory of Esophageal Cancer Prevention & Treatment; Key Laboratory of Henan Province For Drug Quality and Evaluation; Institute of Drug Discovery and Development; School of Pharmaceutical Sciences, Zhengzhou University Zhengzhou China

**Keywords:** autoimmune pathologies, cancer, phosphorylation, protein kinase C family, protein kinase C isoforms, therapeutic targets

## Abstract

The protein kinase C (PKC) family represents pivotal regulators in cellular signaling, whose dysregulation has been implicated in diverse human diseases, including cancer, neurodegenerative disorders, and metabolic syndromes. PKCs transduce extracellular signals through lipid‐mediated activation and controlled subcellular translocation. Their activity is orchestrated by a multistep life cycle, encompassing constitutive phosphorylation during maturation, second messenger‐dependent activation, and agonist‐driven termination. Despite extensive investigation, critical gaps remain in the isoform‐specific signaling networks and the structural determinants that underlie PKC functional diversity, thereby limiting the development of targeted therapies. In this review, we provide a comprehensive analysis of the PKC family, covering isoform diversity, structural and functional attributes, physiological roles, involvement in disease pathogenesis, therapeutic targeting strategies, as well as current controversies and research challenges. PKCs precisely regulate cell fate through subtype‐specific signaling networks, and their structural plasticity presents unique opportunities for therapeutic intervention. By integrating recent advances from structural biology, disease models, and clinical trials, this review proposes a unified framework of PKC regulation that bridges fundamental biology with translational innovation. Ultimately, it serves as a valuable resource for elucidating the multifaceted roles of PKCs in health and disease, while providing a conceptual basis for the rational design of next‐generation therapeutics.

## Graphical abstract

Ligand–receptor binding activates PLC, which hydrolyzes the membrane lipid PIP_2_, generating the second messenger's IP_3_ and DAG. IP_3_ binding to ER receptors causes Ca^2^⁺ release. Subsequently, PKC are recruited to the membrane, which induces a conformational change in PKC, leading to the full enzymatic activation. Activated PKC then drives cellular responses by phosphorylating downstream substrate proteins.

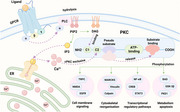

## 1 Introduction

Protein kinase C (PKC) is a family of protein kinases that phosphorylate serine/threonine residues within substrate protein molecules. In 1977, Japanese scientist Nishizuka's team isolated PKC for the first time from rat brain, defined as a serine/threonine kinase that can be activated by calcium ions (Ca^2+^), phosphatidylserine (PS), and diglycerides (DAG) [1, 2]. It can regulate various cell types of essential cellular processes that have been the focus of drug discovery efforts ever since their first identification as the receptor for the tumor promoter phorbol ester in 1982 [3, 4]. With further research, the same team revealed that the tumor promoter phorbol 12‐myristate 13‐acetate (PMA) activates PKC by mimicking endogenous DAG, establishing the “phospholipid–Ca^2+^–DAG” trinity activation model and laying the foundation of the lipid signaling pathway [5]. In the late 1980s, PKCα, β, and γ were cloned for the first time by cDNA library screening, confirming that they are a multigene family [6]. While discovery of nPKC (novel PKC) and aPKC (atypical PKC) expanded the classification of the PKC family in the 1990s.

**FIGURE 1:**
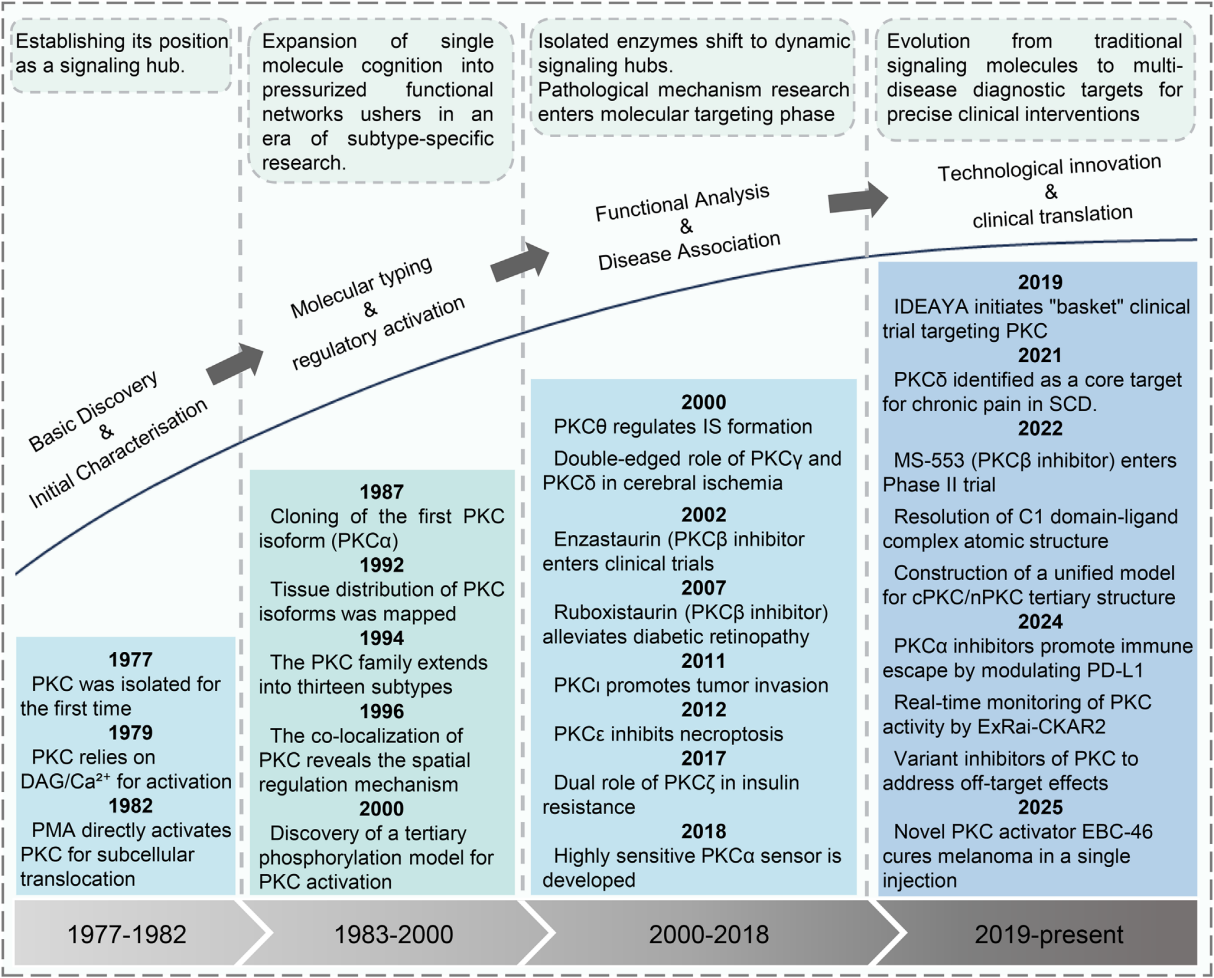
History of the PKC family. The PKC family starts from basic discovery and moves toward clinical translation and disease association through in‐depth mechanism analysis and technological innovation. (Created with BioRender.com.)

The history of the PKC family has gone through four main periods, reflecting the translation from basic to clinical (Figure 1). Despite the large number of positive results accumulated in preclinical studies, progress in clinical translation has been relatively slow. The structural diversity, subtype‐specific functions, and complex roles in pathophysiological processes of the PKC family as central regulators of cellular signal transduction constitute the scientific basis and clinical necessity for writing a comprehensive review.

As research has progressed, PKC biology has taken on a multidimensional complexity, dispersed across a number of fields, including neuroscience, metabolic research, oncology, and immunology. It ranges from molecular structure and subtype‐specific regulatory mechanisms to tissue‐specific functions to role transitions in different disease contexts [7]. To integrate these isolated knowledge modules into a cohesive framework and provide researchers with a panoramic perspective that fosters interdisciplinary collaboration and innovation, this review systematically analyzes the structural and functional characteristics of PKC isozymes. It elaborates on the architecture of the PKC family, its biological roles, and associated pathologies, while delineating the mechanisms of action and pathways of relevant inhibitors through both preclinical and clinical investigations. This synthesis aims to serve as a comprehensive reference for researchers in neuroscience, metabolic pathology, immunology, and drug discovery. We hope to provide a theoretical framework for researchers in the fields of neuroscience, metabolic disease, immunology, and drug discovery and development and ultimately promote the development of PKC‐targeted precision therapeutic strategies, bringing new breakthroughs in the study of the mechanisms and treatment of complex diseases.

This narrative review synthesized literature through iterative searches in multidisciplinary databases (e.g., PubMed, Web of Science, Scopus, Embase, Cochrane Library) using core keywords such as “pkc” and “review”. The search focused on publications from 1977 to 2025, prioritizing seminal works and recent breakthroughs (post‐2020). During the composition of the clinical trials section, the ClinicalTrials.gov database (https://clinicaltrials.gov) was thoroughly consulted to investigate the latest advancements in this domain, which is a globally recognized clinical trial registry managed by the United States National Institutes of Health, which aggregates over 450,000 studies from 220 countries. At the same time, we also referred to the World Health Organization (WHO), International Clinical Trails Registry Platform (ICTRP), EU Clinical Trials Register (EUCTR), and other databases. Given the functional heterogeneity of PKC family members, this review cannot outline all of their regulatory mechanisms. However, by integrating recent groundbreaking studies, this paper systematically comprehends the key roles of PKCs in disease signalling pathways, providing a multidimensional cross‐cutting perspective for understanding their pathophysiological functions, which may provide a new logical framework and research paradigm for precision intervention strategies targeting PKC subtypes.

## 2 Structural Domains of the PKC Family

Through molecular cloning and enzymatic analyses, the mammalian PKC family has now been confirmed to consist of 13 related isoforms, separated into three different classes based on divergent regulatory domains of isoform structure and the ability to respond to second messenger requirements: (I) DAG‐ and Ca^2+^‐dependent classical isoforms (cPKC: α, βI, βII, and γ); (II) DAG‐dependent novel isoforms (nPKC: δ, ε, η, and θ); (III) DAG‐ and Ca^2+^‐independent atypical isoforms (aPKC: λ, ι, and ζ) [8, 9]. All PKC isozymes contain N‐terminal regulatory and C‐terminal catalytic structural domains, each of which plays a specific role in enzyme activation, subcellular localization and functioning [6]. These kinases contain a highly conserved catalytic domain (consisting of motifs required for adenosine triphosphate [ATP]/receptor binding and catalysis) and a regulatory domain that maintains the enzyme in its inactive form.

The regulatory domain of PKC is located at the NH_2_ terminus of the protein and contains an autoinhibitory pseudo receptor domain and two discrete membrane targeting modules (C1 and C2). As the primary DAG sensor for PKC, the C1 structural domain is present in all cPKC and nPKC isoforms and is degraded or absent in aPKC [8]. The C1 structural domain consists of sequences in tandem about 50 residues long, each with six galactose‐binding sites and two histidine residues, forming a cysteine‐rich bicyclic structure stabilized in its three‐dimensional conformation by two zinc ions, which function as a DAG‐/PMA‐binding motif [10]. The ligand‐binding groove in C1 structural domain has a unique two‐sided chemistry: a hydrophilic surface at the bottom, which can form hydrogen bonds with the glycerol backbone of DAG, and a hydrophobic surface at the top, which is suitable for accommodating the fatty acid chain of DAG. This “amphiphilic” design allows the C1 domain to accurately recognize DAG molecules in the lipid environment [11]. In the inactivated state, the C1 domain is highly dynamic, searching for ligands on the membrane surface like Pac‐Man [12]. Once bound to DAG, it is immediately conformationally stable and tightly wraps the ligand molecule through a network of hydrophobic interactions and hydrogen bonds [13]. The structures of the C1–ligand complexes, resolved by X‐ray crystallography and nuclear magnetic resonance techniques, show that the C1 structural domains of the different PKC isoforms, although conserved as a whole, differ subtly in the geometry of the binding pockets and the distribution of surface charges [14].

The C2 structural domain found only in cPKC forms a typical β‐sandwich fold and contains multiple calcium‐binding sites, which are key modules for calcium‐dependent membrane targeting [15]. When cytoplasmic Ca^2+^ concentration increases, Ca^2+^ binds to the C2 domain, inducing a conformational change that exposes a positively charged membrane‐bound surface [16]. It specifically recognizes PS in the inner leaflet of the plasma membrane, enabling the directed recruitment of PKC to the plasma membrane [17]. This process creates spatial proximity for the subsequent binding of DAG to the C1 structural domain. Although C2‐like sequences are present in nPKC, they cannot be activated by Ca^2+^ due to the lack of key calcium‐liganding acidic residues. It can be maximally activated by agonists that promote DAG accumulation or by PMA without the need for calcium [9].

The regulatory structural domains of PKC are key modules for their sensing of the cell membrane lipid environment and second messengers, and differences in regulatory structural domains between isoforms determine their specific responses to activation signals. The C‐terminal catalytic structural domain of PKC contains two highly conserved functional modules: C3 (ATP‐binding domain) and C4 (substrate‐binding domain), which together form the core of kinase activity. The C3 structural domain contains the typical Gly–X–Gly–X–X–Gly–Lys motif that forms the ATP‐binding pocket. This structural domain is responsible for binding ATP and transferring its γ‐phosphate group in a directed manner to serine/threonine residues of substrate proteins. In classical PKC (cPKC) and nPKC, the C3 structural domain contains a unique hinge region that can be regulated by phosphorylation. The newly synthesized PKC molecules require 3‐phosphoinositide‐dependent protein kinase‐1 (PDK1)‐catalyzed phosphorylation (located in the activation loop) to gain full catalytic activity.

The C4 structural domain contains a conserved catalytic loop (HRD motif) and a substrate recognition groove that determines the specificity of PKC for substrate proteins. The C4 structural domains of different PKC isoforms differ significantly, which explains their selectivity for substrate proteins. For example, PKCα prefers substrate sequences containing basic residues, whereas PKCδ tends to recognize motifs containing hydrophobic residues.

**FIGURE 2:**
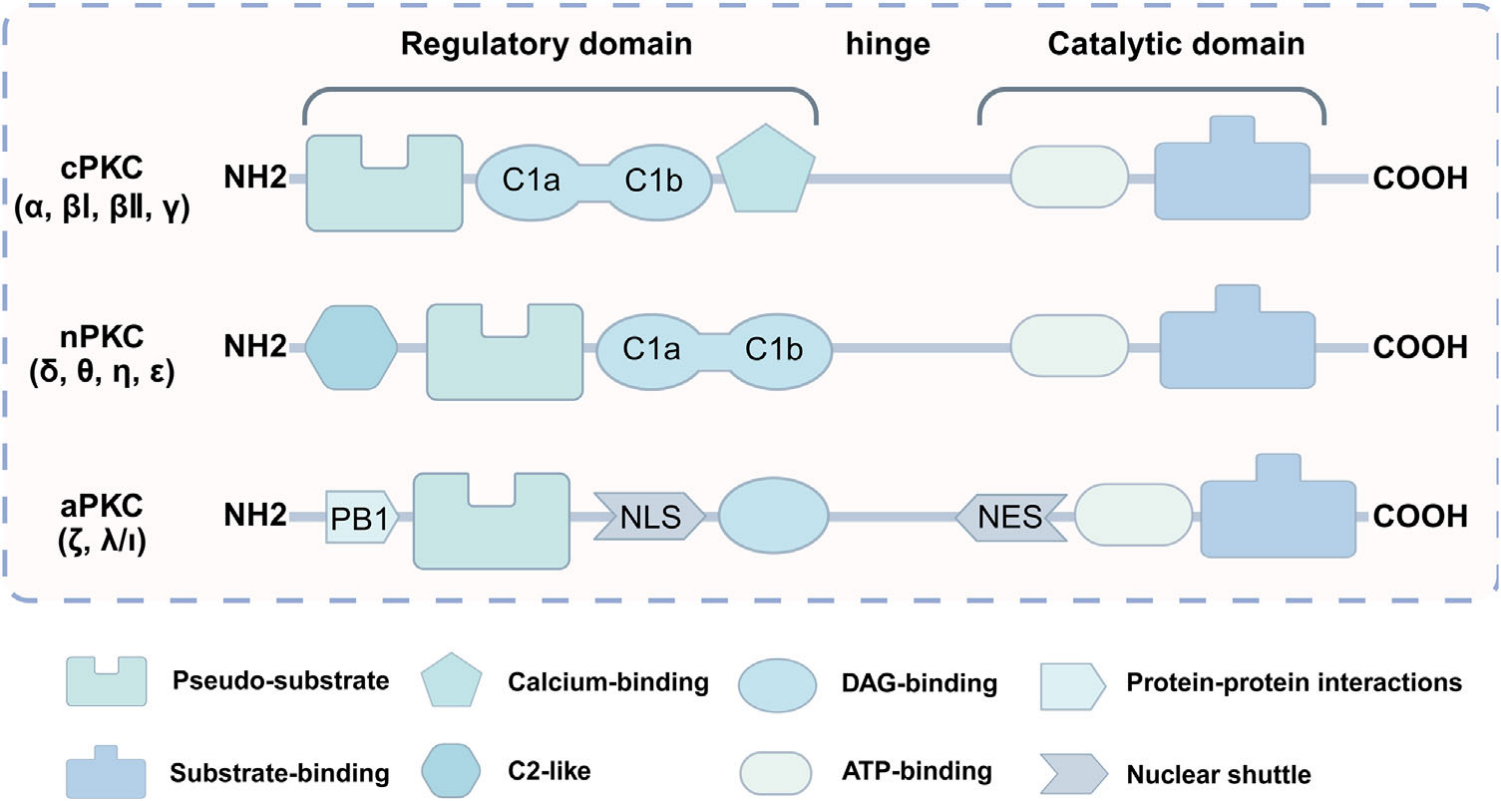
Schematic of primary structures of protein kinase C family members showing domain composition. The PKC family members are divided into three classes (cPKC: α, βI, βII, γ; nPKC: δ, θ, η, ε; aPKC: ζ, λ/ι), each of which shares similar structural domains. (Created with bioRender.com.)

In short, cPKC contains four conserved structural domains (C1–C4), whose activation is strictly dependent on Ca^2+^, PS, and DAG [7, 18]. In the resting state, PKC exists in a “closed” conformation, with the N‐terminal pseudo substrate sequence (located in the regulatory domain) occupying the substrate‐binding groove in the C4 structural domain, keeping the enzyme inactivated [19]. The amino acid sequence of this pseudo‐substrate is similar to that of the real substrate of PKC, but with serine/threonine replaced by glycine at key positions [17]. When the cell is stimulated, second messengers (e.g., DAG, Ca^2+^) bind to the regulatory domain, triggering a conformational change that releases the catalytic domain, leading to kinase activation [20]. For cPKC, the C2 structural domain first binds Ca^2+^, which guides the enzyme to bind to the PS‐containing plasma membrane [21]. The membrane‐bound C1 structural domain captures the DAG, which releases the pseudo‐substrate from autoinhibition, and then the catalytic structural domain is fully exposed, which is phosphorylated to obtain a stable conformation, and the activated PKC phosphorylates the downstream substrate, initiating the signaling transduction [22]. For nPKC, the order of the C1 and C2 structural domains changes along the linear sequence of the protein relative to the cPKC. Its activation does not depend on calcium signaling, but rather on direct binding of DAG through the C1 structural domain to achieve membrane recruitment and autoinhibition release [23]. In contrast to cPKC and nPKC, aPKC lack the C2 structural domain and retain only a portion of the C1 structural domain, and are therefore insensitive to DAG, phosphatidyl myristate, and Ca^2+^, and are activated mainly by protein–protein interactions (PPIs) (e.g., PB1 structural domain‐mediated dimerization) or phosphatidylinositol derivatives (e.g., PIP_3_) [24] (Figure 2). These differences provide a theoretical basis for designing subtype‐selective drugs [25].

## 3 Multiple Functions of PKC

Before membrane translocation, the PKC family must undergo phosphorylation modification to gain full activity. This process occurs through three separate phosphorylation events: a conserved threonine residue in the activation loop (e.g., Thr500 of PKCβII) must be phosphorylated by the upstream kinase PDK1, and autophosphorylation on the TM (Turn motif) stabilizes the kinase core structure (e.g., Thr641 of PKCβII), and phosphorylation of the hydrophobic site at the carboxyl terminus can provide a mooring site for PDK‐1 (e.g., Ser660 of PKCβII) [26].

Differences in sequence homology led to differences in phosphorylation sites across isoforms, which reflect isoform‐specific regulatory mechanisms to some extent. Upon PKC activation, PKC translocases from the cytoplasm to the cell membrane or organelle membrane, where it interacts with specific anchoring proteins and thus localizes to specific subcellular regions to perform its function. This multilevel regulatory mechanism ensures that the cell is able to precisely control PKC activity in response to a variety of extracellular signaling stimuli [27].

**FIGURE 3:**
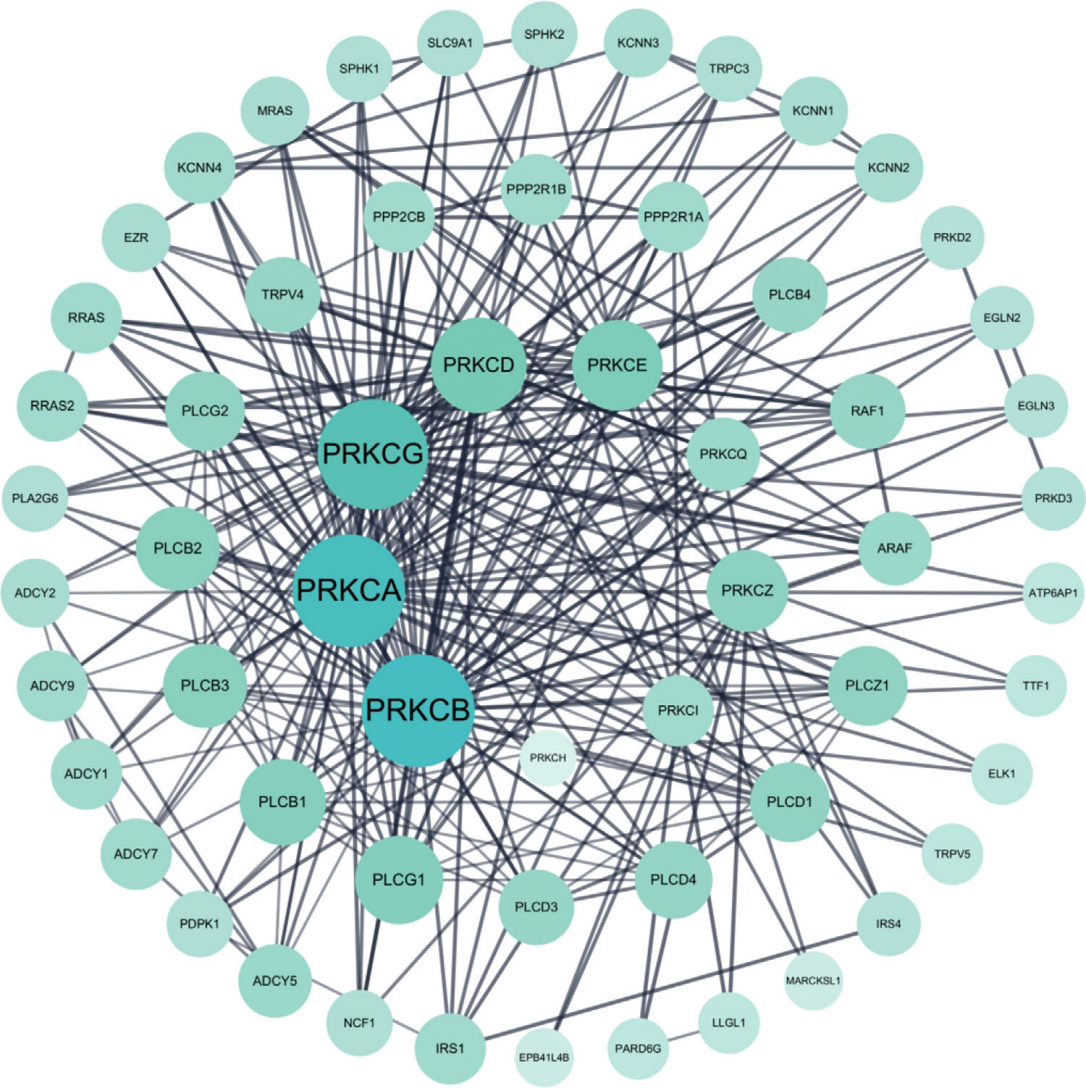
Interaction map of the hub genes. Protein interaction networks of PKC family with classical signaling pathways analyzed by bioinformatics. (Created with bioRender.com.)

As shown in Table 1, the different PKC isoforms showed distinct tissue‐specific distribution patterns in terms of subcellular localization. Among them, PKCα, PKCδ, and PKCζ are widely distributed throughout the body. PKCβI/βII are mainly distributed in the brain and non‐neural tissues, while PKCγ is central nervous system (CNS) specific, especially highly expressed in the hippocampus, cerebellum, and cerebral cortex. PKCε is mainly distributed in the brain and non‐neural tissues. PKCη has lung, skin, and myocardial specificity. PKCθ is mainly expressed in T lymphocytes, platelets, and skeletal muscle cells, and dominates T‐cell activation in the immune system. PKCι/λ is mainly distributed in the ovary, testis, and pancreatic islet cells. As shown in Figure 3, the PKC family lies at the crossroads of many signaling pathways and is involved in a wide range of G protein‐coupled receptors and other growth factor‐dependent cellular responses, playing a very important role in the cell body [28]. In the following section, the functional characteristics of each subtype are described separately from the subtype.

**TABLE 1:** The PKC family sources and tissue specificity.

PKC subtype	Year	Source	Technical	Functionality	Tissue specificity
PKCα	1986	Bovine brain cDNA library	cDNA cloning, sequencing	Cell proliferation, migration	Wide spread
PKCβI/βII	1987	Rat brain cDNA library	Homology screening	Angiogenesis, inflammatory response	Brain and endocrine organs
PKCγ	1987	Rat brain cDNA library	Low stringency hybridization screening	Neuron‐specific expression involved in learning and memory	Central nervous system
PKCδ	1988	Rat fibroblast cDNA library	PCR amplification and library screening	Proapoptotic function with a complex role in cancer	Wide spread
PKCε	1988	Rat brain cDNA library	Primer PCR	Involved in myocardial protection, neuroplasticity	Brain, lungs, oval cells
PKCζ	1993	Human placenta cDNA library	Primer PCR	Involved in cell polarity and survival signaling	Wide spread
PKCη	1994	Human epithelial cell cDNA library	Primer PCR	Key factors in skin and epithelial cell differentiation	Lungs, skin, heart muscle
PKCθ	1994	T‐lymphocyte cDNA library	Differential hybridization screening	T cell receptor signaling core kinase	Skeletal muscle, T cells
PKCι	1993	Human brain DNA library	Homology screening	Cell polarity establishment and tumorigenesis correlate	Ovary, testis, islet cells
PKCλ	1994	Mouse embryo cDNA library	Homology screening	Cell polarity establishment and tumorigenesis correlate	Ovary, testis, islet cells

### 3.1 PKCα

PKCα, the most widely distributed classical isoform, has been implicated in cell proliferation, apoptosis, proliferation, motility, and inflammation. The alterations in cellular responses that it induces are not intrinsic properties, but are regulated through dynamic interactions with cell‐type‐specific factors [29, 30]. PKCα phosphorylates cytoskeleton‐associated proteins and influences the dynamics of actin polymerization, stress fiber formation, and adhesion patches, thereby regulating cell morphology and motility [31, 32]. It also regulates cell adhesion to the extracellular matrix and influences integrin activity and signaling [33, 34]. It has been demonstrated that nuclear translocation of PKCα is associated with cell cycle arrest and erythroid differentiation in myelodysplastic syndromes [35]. Studies have shown that PKCα is antiproliferative and differentiation‐inducing in some normal tissues (e.g., regenerating epithelium) and proproliferative in other tissues (e.g., cells of the hematopoietic system, smooth muscle cells), highlighting its dual role in tumors [36]. The overexpression and activation state of PKCα also correlate with proproliferative signaling when it is present, which often associates it with tumor proliferation, invasion, metastasis, angiogenesis, chemoresistance, and poor prognosis [37]. PKCα expression level was positively correlated with the tumor node metastasis stage of the tumor, and the higher the degree of malignancy, the higher the PKCα level, indicating that it is closely related to the growth and invasive ability of tumor cells [38]. In contrast, S‐phase‐specific activation of PKCα induced senescence in non‐small cell lung cancer (NSCLC) cells, and similarly, inhibition of PKCα promoted breast cancer immune escape by maintaining the stability of programmed cell death 1 ligand 1 (PD‐L1) [39, 40]. This inhibitory and promotional effect on tumors reflects the tissue‐specific and context‐dependent nature of PKCα.

In addition to its dual role in oncology, PKCα also regulates vascular function and generation. In vascular endothelial cells and smooth muscle cells, PKCα can be activated by a variety of vasoactive substances and thus participates in the regulation of vasoconstriction, vascular permeability, endothelial cell migration, proliferation, and survival [41, 42].

In cardiomyocytes, PKCα is involved in the regulation of cardiac repolarization by mediating the inhibition of myocardial rapid delayed rectifier potassium current/human ether‐à‐go‐go related gene (Ikr‐hERG) currents by α_1_A‐adrenergic receptors, thereby prolonging action potential duration [43, 44, 45]. In vascular smooth muscle, PKCα affects vasoconstriction by indirectly phosphorylating myosin light chain 20 (MLC20) and modulating calcium sensitivity [46, 47]. In hemorrhagic shock, activation of PKCα partially restores the sensitivity of shocked blood vessels to norepinephrine and Ca^2+^, improves vascular hyperresponsiveness, and maintains blood pressure and microcirculatory perfusion [46, 48]. In colonic smooth muscle cells, membrane PKCα and transported calpain form a calpain–PKCα–heat shock protein 27 (HSP27) complex to promote smooth muscle contraction [49]. Thus, PKCα plays an important role in angiogenesis and the pathology of vascular diseases (e.g., hypertension, atherosclerosis, diabetic vascular complications).

PKCα also has a central role in the nervous system and metabolism. PKCα binds to adaptor proteins via the PDZ structural domain and directly regulates actin reorganization and pre‐/postsynaptic structural remodeling to promote dendritic spine morphological plasticity [50, 51]. PKCα indirectly causes neuronal atrophy by phosphorylating the α‐amino‐3‐hydroxy‐5‐methyl‐4‐isoxazolepropionic acid receptor (AMPA) receptor subunit GluR2 (Ser880 site), driving it away from synaptic anchoring proteins (e.g., glutamate receptor‐interacting protein/AMPA receptor‐binding protein [GRIP/ABP]) and affecting receptor endocytosis and synaptic plasticity [52]. Recent studies have shown that PKCα functionally acquired mutation (M489V) accelerates synaptic loss, reduces dendritic spine density, and cognitive decline in Alzheimer's disease (AD) model mice, and inhibition of PKCα activity emerges as a potential therapeutic strategy [53]. In a gallstone model, PKCα activation down‐regulates the bile acid transporter protein ATP‐Binding cassette subfamily B member 11 (ABCB11), which is directly associated with disturbed bile metabolism and promotes cholesterol crystallization and gallstone formation [54]. PKCα phosphorylates insulin receptor substrate 1 (IRS‐1), inhibits insulin receptor signaling in negative feedback, reduces glucose transporter type 4 (GLUT4) membrane translocation and glucose uptake, and is involved in insulin resistance [55]. It can also activate sterol regulatory element‐binding protein‐1c (SREBP‐1c), promote fatty acid synthase expression, and increase hepatic lipid accumulation; it also inhibits Akt (PKB) phosphorylation and interferes with insulin metabolic pathways [55].

PKCα is also involved in the differentiation of various cell types (e.g., keratinocytes, osteoblasts, neurons, immune cells), in the regulation of inflammatory factors (e.g., tumor necrosis factor‐alpha [TNF‐α], interleukin‐6 [IL‐6], and reactive oxygen species [ROS]), and in the regulation of glucose transport, glycogen metabolism, and lipid metabolism (in the context of insulin signaling pathways)[56, 57].

### 3.2 PKCβI/II

PKCβ is divided into two isoforms, PKCβI and PKCβII, by selective splicing of the PRKCB1 gene, which play key regulatory roles in a variety of physiological and pathological processes, particularly in metabolic disease complications, tumorigenesis, inflammatory responses, and cell death pathways [58, 59, 60]. PKCβI is mainly localized in the cytoplasm and is involved in homeostatic regulation (e.g., neuroprotection, cell adhesion), whereas PKCβII is mainly enriched in the cell membrane and Golgi and dominates the stress response (e.g., lipid peroxidation perception, amplification of inflammatory signals) [61, 62]. As a core regulator of metabolic diseases and their complications, PKCβ is specifically induced to be expressed in white adipose tissue (WAT) under a high‐fat diet, leading to obesity, insulin resistance, and fatty liver by disrupting mitochondrial function and regulating the p66Shc signaling pathway [63]. Experimental studies have shown that PKCβII activation by PI3K phosphorylates its substrate myristoylated alanine‐rich C‐kinase substrate (MARCKS), which promotes cytoskeletal reorganization through the release of phosphatidylinositol 4,5‐bisphosphate (PIP2) and activation of phospholipase D1, thereby enhancing GLUT4 membrane translocation [64, 65]. In addition, some PKC isoforms (e.g., PKCθ) can indirectly regulate GLUT4 function by inhibiting the PI3K/Akt pathway through serine phosphorylation of IRS‐1, but this mechanism has not been clarified in PKCβII [66, 67]. At the same time, PKCβ is activated by hyperglycemia and free fatty acids in pancreatic β‐cells, phosphorylating the transcription factor pancreatic and duodenal homeobox 1 (PDX‐1) (a key regulator of insulin genes), leading to a reduction in the nuclear translocation of PDX‐1 and inhibition of the transcription of insulin genes (e.g., Insulin 2) [63]. In patients with type 2 diabetes mellitus, the magnitude of glucose fluctuation is positively correlated with PKCβ1 expression, which promotes vascular endothelial damage and platelet aggregation and accelerates atherosclerosis through the up‐regulation of E‐selectin, von Willebrand Factor, and CD62p [68, 69].

PKCβ is associated with both diabetic complications, nephropathy and retinopathy. It can upregulate transforming growth factor beta 1 (TGF‐β1) via the DAG–PKC pathway and promote Ras homolog gene family member A (RhoA) activation as well as fibronectin deposition in glomerular mesangial cells, subsequently leading to basement membrane thickening, glomerulosclerosis, microalbuminuria, and renal fibrosis [70, 71]. PKCβII senses lipid peroxidation signals and phosphorylates the T328 site of acyl coenzyme A synthetase long‐chain family member 4 (ACSL4) to increase the synthesis of phospholipids containing polyunsaturated fatty acids, thereby amplifying the lipid peroxidation response, resulting in a positive feedback of “lipid peroxidation–PKCβII–ACSL4” axis, which ultimately induces iron‐dependent cell death [72]. In diabetic microvascular complications, PKCβ mediates increased retinal vascular permeability, promotes neovascularization, and contributes to abnormal blood flow, representing a key mechanism in diabetic retinopathy [73, 74]

This same mechanism of involvement in angiogenesis through modulation of the vascular endothelial growth factor (VEGF) signaling pathway allows PKCβ to provide nutritional support for tumor growth in the tumor microenvironment [75, 76]. In lung cancer tissues, both mRNA and protein expression levels of PKCβI are significantly elevated compared with adjacent nontumorous tissues and benign pulmonary lesions. This overexpression accelerates tumor progression by suppressing apoptosis and driving cell cycle advancement [77, 78]. PKCβII potentiates tumor growth (e.g., in gastric and colorectal cancers) by activating the glycogen synthase kinase 3 beta (GSK3β) signaling axis, which enhances cellular proliferation and invasiveness [79, 80]. In chronic myeloid leukemia cell K562, silencing PKCβII inhibits proliferation and blocks G1 phase, suggesting a dependence on the extracellular regulated protein kinases (ERK) pathway to maintain tumor growth [80]. Functioning as a coactivator of the androgen receptor (AR), PKCβII phosphorylates histone H3 threonine 6 (H3T6). This phosphorylation blocks demethylation of histone H3 lysine 4 (H3K4) by lysine‐specific demethylase 1, thereby sustaining an active chromatin configuration at AR target gene promoters and propelling prostate cancer progression [81]. However, H3T6 phosphorylation is still subject to some controversy due to the lack of consistent validation in animal models and clinical samples.

PKCβ exhibits neuroprotective functions in neuropsychiatric disorders through distinct mechanisms, while it is necessary for amygdala‐dependent cues and situational fear conditioning [82]. By stabilizing fat mass and obesity‐associated protein, PKCβ removes m⁶A modifications on peroxisome proliferator‐activated receptor gamma coactivator 1‐alpha mRNA to enhance its stability. This leads to transcriptional activation of uncoupling protein 1 (UCP1), suppression of neuronal apoptosis, and amelioration of autism‐like behaviors [83]. PKCβ critically regulates D2autoR‐activated dopamine transporter (DAT) transport and dopaminergic signaling, emerging as a potential drug target for correcting abnormal extracellular dopamine levels in diseases such as drug addiction and schizophrenia [84]. PKCβII is highly expressed in the basolateral amygdala nucleus, where it regulates fear memory consolidation [82].

The two PKCβ isoforms exert opposing effects in cardiomyocytes: PKCβI demonstrates cardioprotective potential, whereas PKCβII exacerbates inflammation by inducing autophagy dysregulation and oxidative stress [85]. Specifically, PKCβII activates the NOD‐like receptor family, pyrin domain containing 3 inflammasome, promoting caspase‐1 cleavage and IL‐1β maturation/secretion, ultimately triggering pyroptosis in cardiomyocytes [86]. Specific modulation of PKCβ isoforms is essential to achieve disease treatment. Based on the functional differences between the two isoforms of PKCβ, specific modulation of the isoforms is essential for the treatment of the disease.

### 3.3 PKCγ

As a neuron‐specific kinase, PKCγ maintains homeostasis in the CNS through three core functions: amplification of pain signaling, modulation of synaptic plasticity, and fine control of motor coordination. Dysfunction of PKCγ directly contributes to pain hypersensitivity (e.g., neuropathic pain), movement disorders (e.g., spinocerebellar ataxia type 14), and cognitive decline (e.g., memory impairment associated with rapid eye movement sleep deprivation) [87, 88].

PKCγ, highly expressed in spinal cord dorsal horn lamina II–III interneurons, phosphorylates the NR1 subunit of N‐methyl‐d‐aspartate receptors (NMDARs), which relieves the Mg^2+^ block, promotes Ca^2+^ influx, and enhances neuronal excitability [89]. Notably, the influx of Ca^2+^ further activates PKCγ, establishing a “PKCγ–NMDAR” positive feedback axis, which persistently amplifies nociceptive signaling [89]. Under physiological conditions, the excitability of PKCγ‐expressing interneurons is tonically inhibited. Following nerve injury, glycinergic neurons are suppressed or eliminated, followed by the disinhibition of the PKCγ interneurons, which contributes to the development of mechanical allodynia/hyperalgesia [90, 91, 92].

As a core regulator of synaptic plasticity, PKCγ enriched in the hippocampal CA1 region directly phosphorylates the Ser831 site of the AMPA receptor subunit GluA1 during the early phase of long‐term potentiation (LTP). This phosphorylation enhances the receptor's sensitivity to glutamate and increases channel open probability, thereby elevating synaptic transmission efficiency [93, 94]. Concurrently, PKCγ phosphorylates the N‐methyl‐D‐aspartate receptor subunit 2B (NR2B) subunit of NMDARs, relieving the Mg^2+^ block and augmenting Ca^2+^ influx. This cascade activates the downstream calcium/calmodulin‐dependent protein kinase II–ERK signaling axis, driving the transition from early‐phase LTP (E‐LTP) to late‐phase LTP (L‐LTP) [95, 96]. In L‐LTP, PKCγ phosphorylates components of the mechanistic target of rapamycin complex 1 (mTORC1) pathway (e.g., mammalian target of rapamycin [mTOR] or Raptor), promoting de novo protein synthesis (e.g., PKMζ, Arc) within dendritic spines to sustain LTP maintenance [97]. Additionally, PKCγ phosphorylates specific serine sites in synapsin I, releasing its anchoring effect on synaptic vesicles. This increases the probability of glutamate release and modulates the conversion of short‐term plasticity to LTP [98, 99].

In cerebellar Purkinje cells, PKCγ modulates motor output by regulating key ion channels. Specifically, PKCγ phosphorylates and suppresses the open probability of large conductance calcium‐activated potassium channel channels by targeting their α‐subunits. This suppression increases dendritic membrane resistance, ensuring the fidelity of climbing fiber (CF) signal propagation to the soma and thereby regulating motor learning [100, 101]. Crucially, the importance of PKCγ in the motor system is undoubtedly supported by the fact that developmental loss of PKCγ leads to the persistence of aberrant multiple synaptic contacts from CFs, resulting in adult‐onset motor ataxia [102].

### 3.4 PKCδ

PKCδ exhibits a unique dual role in the regulation of apoptosis, with its functional direction highly dependent on cell type, the nature of the stimulus, and subcellular localization [103]. Under the action of DNA‐damaging agents (such as ultraviolet radiation or chemotherapeutic drugs), PKCδ is phosphorylated by tyrosine kinases like cellular‐Abelson gene (c‐Abl) at residues Tyr64 and Tyr187 [104]. Phosphorylated PKCδ forms a positive feedback loop with c‐Abl: c‐Abl activates PKCδ, while PKCδ, in turn, enhances c‐Abl activity by inhibiting the tyrosine phosphatase SHP‐1 [105]. Activated PKCδ is cleaved by caspase‐3 into a constitutively active 40 kDa catalytic fragment, which can translocate into the nucleus based on its nuclear localization signal [106, 107]. Within the nucleus, PKCδ directly binds to and activates the transcription factor Btf (Bcl‐2‐associated transcription factor 1, BCLAF1). Btf specifically binds to the core promoter element of tumor protein P53 (TP53), significantly elevating TP53 transcription levels, thereby inducing cell cycle arrest or apoptosis [105, 108]. In contrast, PKCδ displays antiapoptotic properties in immune cell tolerance (e.g., B‐cell autoantigen response) in response to cytokine receptor signaling (e.g., insulin‐like growth factor 1 receptor) or stimulation by certain growth factors [109].

PKCδ is crucial for neuronal migration, synapse formation, and neurotransmitter regulation within the nervous system. Early research demonstrated that upon activation by brain‐derived neurotrophic factor (BDNF), PKCδ directly phosphorylates the p35 protein. This phosphorylation delays p35 degradation, thereby maintaining the activity of the cyclin‐dependent kinase 5 (CDK5)/p35 complex. This process promotes radial migration and laminar positioning of newborn neurons in the cerebral cortex, ensuring their proper localization [110]. PKCδ regulates the internalization of the DAT and downregulates its membrane expression, consequently influencing dopamine release and reuptake [103, 111]. In Parkinson's disease (PD) models, excessive activation of PKCδ promotes oxidative stress and mitochondrial damage, facilitating the apoptosis of dopaminergic neurons in the substantia nigra pars compacta [112]. Conversely, in schizophrenia, dysregulation of PKCδ may contribute to the pathogenesis through aberrant D2 receptor signaling [113].

PKCδ exhibits a dual‐edged role in diabetes, acting as a central mediator of β‐cell apoptosis and insulin resistance while also serving as a physiological regulator of α‐cell function and metabolic processes in certain tissues. In a high‐fat environment, free fatty acids upregulate TRB3 expression via endoplasmic reticulum (ER) stress, leading to PKCδ activation and its nuclear translocation. This promotes β‐cell apoptosis by upregulating the proapoptotic protein BCL2‐associated X protein and activating the c‐Jun N‐terminal kinase (JNK) pathway [114]. PKCδ mediates the connection between ER stress and impaired insulin signaling [115]. First, PKCδ interferes with insulin signal transduction by phosphorylating IRS at Ser307. This inhibits the PI3K/AKT pathway, resulting in increased hepatic gluconeogenesis (via fork head box protein O1 activation) and reduced glycogen synthesis (via GSK‐3 inhibition) [116, 117]. PKCδ negatively feeds back to inhibit the insulin receptor by activating the mTORC1/ribosomal protein S6 kinase pathway. This reduces GLUT4 translocation to the plasma membrane, diminishing glucose uptake efficiency [118, 119]. Under obese conditions, aberrant activation of PKCδ in adipose tissue promotes the secretion of inflammatory cytokines (e.g., IL‐6), indirectly exacerbating systemic insulin resistance [119]. Besides, PKCδ is activated in response to arginine stimulation through phosphorylation at Thr505, promoting glucagon secretion [120]. PKCδ‐expressing neurons in the central amygdala sense peripheral leucine deficiency. They activate the general control nonderepressible 2/activating transcription factor 4 signaling pathway, enhancing neuronal activity. This subsequently activates the sympathetic nervous system and releases norepinephrine, which acts on β3‐adrenergic receptors in WAT. This interaction induces UCP1 expression, promoting WAT browning (conversion into thermogenic beige‐like adipocytes) and increasing energy expenditure [121]. PKCδ in hypothalamic microglia is activated via Zinc finger DHHC‐type palmitoyl transferase 5 (ZDHHC5)‐mediated palmitoylation. This activation triggers neuroinflammation (release of TNF‐α, IL‐6), damaging neighboring thyrotropin releasing hormone (TRH) neurons. Reduced TRH secretion leads to decreased thyroid hormone synthesis, which in turn suppresses hepatic fatty acid oxidation (via downregulation of carnitine palmitoyl transferase 1A and peroxisome proliferator‐activated receptor alpha), promoting lipid accumulation [122].

PKCδ exhibits cell‐type‐specific regulatory functions within the immune system. In B cells, it negatively regulates proliferation and participates in self‐antigen tolerance [123]. In T cells, PKCδ influences the activation threshold and differentiation [124]. In neutrophils and eosinophils, PKCδ phosphorylates the p47phox subunit, facilitating the assembly of the nicotinamide adenine dinucleotide phosphate oxidase complex and enhancing the ROS burst for pathogen clearance [125]. This functional diversity underscores PKCδ’s role in the fine‐tuning of immune homeostasis.

### 3.5 PKCε

PKCε serves as a pivotal node in regulating metabolic signaling, neuronal survival pathways, and oncogenic signaling networks. It maintains physiological homeostasis while driving pathological progression across multiple diseases. Mechanistically, sn‐1,2‐DAG activates PKCε, facilitating its translocation to the plasma membrane. Membrane‐localized PKCε phosphorylates Thr1160 on the insulin receptor kinase, impairing insulin signaling cascades (e.g., PI3K/AKT). This disruption suppresses glycogen synthesis and promotes hepatic gluconeogenesis, ultimately contributing to systemic insulin resistance and the pathogenesis of type 2 diabetes [126, 127].

In the nervous system, PKCε exerts neuroprotective effects by activating nicotinamide phosphoribosyl transferase (Nampt) to maintain NAD^+^ levels, enhancing mitochondrial function and reducing ROS production [128, 129, 130]. Although sustained activation of PKCε induces mitochondrial division and membrane potential hyperpolarization, this process may be inhibited in neurons [131]. For example, in neuroprotective models, PKCε reduces apoptosis due to excessive division by blocking the activation of dynamin‐related protein 1, a key mitochondrial division protein [132]. PKCε enhances the enzymatic activity of aldehyde dehydrogenase 2 (ALDH2) by directly phosphorylating specific residues of ALDH2, such as the Ser/Lys site. ALDH2 protects mitochondrial function and reduces apoptosis through metabolism of lipid peroxidation products, such as 4‐hydroxynonenal, and by inhibiting the formation of adducts with mitochondrial proteins [133]. Notably, PKCε exhibits a functional duality: it serves a protective role in acute injury but transitions to a prodegenerative factor in chronic neurodegeneration. This paradigm shift is critically governed by the duration of PKCε activation and tissue microenvironmental cues [134]. Mechanistically, PKCε modulates BDNF and its receptor tropomyosin receptor kinase B (TrkB), inhibiting pathological ion channel hyperactivation and attenuating apoptotic cascades [135, 136]. At the same time, this pathway also activates the ERK–cAMP response element‐binding protein pathway (CREB) pathway, which promotes synaptic plasticity and protects against learning memories [133]. PKCε phosphorylates the Ser839 site of metabotropic glutamate receptor 5, leading to its aberrant endocytosis and degradation, disrupting calcium oscillatory homeostasis and inducing calcium overload in motor neurons [137, 138].

PKCε is overexpressed in multiple malignancies, particularly NSCLC [139, 140]. Mechanistically, PKCε drives oncogenesis by activating the C‐X‐C motif chemokine ligand 13 (CXCL13)–C‐X‐C motif chemokine receptor 5 (CXCR5) axis alongside ERK1/2 and nuclear factor kappa B (NF‐κB) pathways, thereby promoting cellular proliferation and suppressing apoptosis [141, 142, 143]. Meanwhile, PKCε promotes cell motility and metastasis (e.g., membrane ruffle formation, matrix protease secretion) through Rac1 activation to facilitate NSCLC cell invasion [144]. In addition, the above mechanisms may indirectly promote inflammation by stimulating the secretion of proinflammatory cytokines, including IL‐6 and TNF‐α, ultimately creating a microenvironment conducive to tumorigenesis [145, 146]. Notably, the PKCε‐specific activator ε‐PKC receptor for activated C kinase peptide demonstrates prosurvival functions in hematological contexts by antagonizing TNF‐related apoptosis‐inducing ligand‐induced apoptosis in HL‐60 cells and suppressing monocytic differentiation through downregulation of CD14 expression [140, 147].

PKCε serves as a multifunctional regulator in immune modulation and synaptic physiology. In B cells, it orchestrates actin cytoskeletal remodeling and MLC phosphorylation, amplifying antigen‐directed traction forces (10–20 nN) to facilitate B cell receptor micro cluster coalescence and immunological synapse formation, thereby enhancing antigen‐presentation efficiency [137, 138, 148]. In the inflammatory signaling cascade, PKCε is recruited to the endosomal membrane by toll‐like receptor 4 (TLR4), which phosphorylates TRIF‐related adaptor molecule (TRAM) and triggers the TANK‐binding kinase 1 (TBK1)–interferon (IFN) regulatory factor 3 cascade response, driving the secretion of antiviral cytokines such as IFN‐β. This process is parallel but independent of the NF‐κB pathway, and together they promote proinflammatory cytokine secretion. [149]. Beyond these roles, PKCε exhibits specific enrichment at motor nerve terminals, where it is activated by synaptic activity via BDNF–TrkB signaling. This triggers phosphorylation of the MARCKS protein, modulating actin dynamics to potentiate acetylcholine release at neuromuscular junctions [150, 151].

### 3.6 PKCη

PKCη serves as a critical regulator in epithelial and immune cells. By integrating signals related to senescence, barrier function, immunity, and metabolism, it plays a pivotal role in both physiological homeostasis and pathological dysregulation [152].

PKCη primarily promotes stress‐induced senescence (e.g., oxidative damage, chemotherapy) by mediating cell cycle arrest and regulating the senescence‐associated secretory phenotype. Specifically, PKCη upregulates the CDK inhibitors p21Cip1 (and possibly p27Kip1 in specific contexts), leading to inhibition of CDK activity and blockade of the gap 1 phase/synthesis phase (G1/S phase) transition [153]. Furthermore, PKCη selectively enhances the transcription and secretion of IL‐6. It also amplifies senescence signaling through a positive feedback loop by upregulating the IL‐6 receptor, while simultaneously suppressing IL‐8 expression to optimize the senescence‐associated microenvironment [154].

In keratinocytes, PKCη plays a significant role in epithelial barrier formation and differentiation processes [155]. Specifically, research demonstrates that PKCη regulates barrier function by phosphorylating the tight junction protein occludin. More precisely, it targets conserved Thr403/Thr404 residues within the C‐terminal domain of occludin, thereby promoting its integration into the tight junction complex [156]. Beyond its role in barrier formation, PKCη promotes keratinocyte differentiation through dual mechanisms: (1) ras‐like GTPase A binding via the C1 domain drives morphological changes (e.g., actin depolymerization and cell flattening) in a kinase‐independent manner; (2) catalytic activity regulates gene expression of differentiation markers (e.g., TGase1 and involucrin) [157]. This interaction induces actin depolymerization, ultimately driving the morphological transition of keratinocytes from a rounded to a flattened shape—a hallmark of terminal differentiation [158].

PKCη is highly expressed in mature T cells, particularly within the CD8^+^ T cell subset, where it cooperates with PKCθ to regulate TCR signaling and promote antigen‐responsive proliferation of CD8^+^ T cells [159, 160]. Intriguingly, as the only PKC isoform that integrates TCR signaling with tissue‐resident survival, PKCη uniquely governs T cell homeostatic proliferation—a process dependent on self‐antigen recognition, which provides new targets for mucosal immunotherapy [161]. Strikingly, loss of PKCη disrupts the CD4^+^/CD8^+^ T cell balance, leading to an increased CD4^+^ T cell proportion. Conversely, PKCθ deficiency reduces the CD4^+^ proportion. Critically, simultaneous deletion of both PKCη and PKCθ restores normal T cell subset ratios, revealing their antagonistic roles in maintaining T cell population equilibrium [159, 162]. Furthermore, and of significant functional importance, PKCη acts as a key effector molecule for the immunosuppressive function of regulatory T (Treg) cells by physically interacting with the immune checkpoint protein cytotoxic T‐lymphocyte associated protein 4. This interaction is implicated in the blockade of tumor immune surveillance [154, 163].

Remarkably, the functional scope of PKCη extends beyond the previously discussed roles. Within the VEGF signaling pathway, PKCη collaborates synergistically with PKCε to mediate the differentiation of human embryonic stem cells into endothelial cells [164, 165]. Critically, this process involves a self‐amplifying positive feedback loop that enhances VEGF secretion and sustains PKC activation [166, 167]. Furthermore, in the context of innate immunity and hematopoiesis, IFNα/β rapidly activates PKCη phosphorylation. This activation, in turn, induces G0/G1 phase cell cycle arrest and exerts a potent inhibitory effect on colony forming unit‐granulocyte macrophage, thereby suppressing the proliferation of both normal and leukemic myeloid progenitors [153, 154]. Additionally, PKCη contributes significantly to vascular homeostasis by synergizing with PKCβ to optimize endothelial nitric oxide (NO) generation [168, 169].

### 3.7 PKCθ

PKCθ functions as a critical integrator of TCR and CD28 costimulatory signaling, orchestrating T cell immune responses by activating key downstream transcription factors [170]. Following antigen recognition, PKCθ is specifically recruited to the central supramolecular activation cluster (cSMAC) of the immunological synapse, a process critically dependent on PI3K‐mediated Rac activation and subsequent actin cytoskeleton reorganization [171]. Concurrently, TCR engagement triggers phospholipase C‐γ1 to hydrolyze PIP_2_, generating DAG. This lipid second messenger binds the C1 domain of PKCθ, facilitating its translocation from the cytosol to the plasma membrane [172]. Full enzymatic activation is achieved through phosphorylation by the Src‐family kinase lymphocyte‐specific protein tyrosine kinase (Lck), which is recruited and activated by CD28 costimulation [173]. This tightly regulated activation cascade is indispensable for mature T cell activation, proliferation, survival, and the production of essential cytokines such as IL‐2 and IFN‐γ [174]. Downstream, activated PKCθ phosphorylates caspase recruitment domain‐containing protein 11 at Ser564/Ser657, triggering a conformational change that permits B‐cell lymphoma/leukemia 10 (BCL10) recruitment and assembly of the CBM signaling complex (CARMA1–BCL10–MALT1), in coordination with BCL10 ubiquitination to activate NF‐κB [175, 176]. This complex subsequently activates the IκB kinase (IKK) complex, leading to NF‐κB nuclear translocation and transcriptional induction of genes like IL‐2, which drive T cell proliferation. Furthermore, PKCθ activates the activator protein 1 (AP‐1) transcription factor complex independently of the mitogen‐activated protein kinase (MAPK) pathway via phosphorylation of the Ste20‐related proline‐alanine‐rich kinase, promoting Fos/Jun heterodimer formation to regulate proliferation and differentiation genes. Concurrently, PKCθ contributes to nuclear factor of activated t‐cells (NFAT) signaling by influencing calcium mobilization and calcineurin activity, enabling NFAT nuclear translocation [177]. Within the nucleus, NFAT synergistically cooperates with AP‐1 to activate target genes essential for T cell clonal expansion [178].

As the exclusive PKC isoform recruited to the cSMAC of the immunological synapse (IS) following TCR engagement with major histocompatibility complex Class II (MHC‐II), PKCθ exerts a selective regulatory role in T cell differentiation and corresponding immune responses [179]. This selectivity is underscored by studies demonstrating that PKCθ‐knockout mice exhibit tolerance to allergic reactions, autoimmune diseases, and allogeneic immune rejection, while remarkably retaining intact protective immunity against bacterial, viral, and tumor challenges [178]. PKCθ critically governs T cell activation, differentiation, and survival by specifically modulating lineage‐specific T helper (Th) cell development [180]. Notably, investigations into Th2‐type immune responses against allergens and helminth infections, as well as Th17‐mediated experimental autoimmune encephalomyelitis (EAE), have established that PKCθ is indispensable for promoting the differentiation of Th2 and Th17 cells and their subsequent effector functions [181, 182]. In stark contrast to its crucial role in Th2/Th17 biology, PKCθ is largely dispensable for Th1 cell differentiation and function [183]. Moreover, PKCθ expression extends beyond conventional T cells to natural killer (NK) cells, where it is implicated as essential for NK cell‐mediated antitumor surveillance [184]. Crucially, PKCθ also functions as a negative regulator, restraining the differentiation and suppressive function of Treg cells and thereby acting as a physiological counterbalance to prevent excessive immunosuppression [185].

The selective role of PKCθ within the immune system demonstrates significant therapeutic potential for autoimmune diseases such as MS (multiple sclerosis) and rheumatoid arthritis. Beyond this beneficial aspect, PKCθ activation contributes to detrimental outcomes, including the induction of graft‐versus‐host disease (GVHD) and organ damage, while simultaneously promoting posttransplant T cell activation and survival [186]. Moreover, PKCθ exacerbates tumor‐associated bone destruction by driving osteoclast differentiation through activation of the NF‐κB/IL‐1β axis [187]. PKCθ contributes to pathogenesis in two distinct ways. In the hematopoietic system, it promotes CD8^+^ Th1 cell activation and Th1 cell differentiation, triggering the release of proinflammatory cytokines (e.g., TNF‐α and IFN‐γ) that induce apoptosis in hematopoietic stem and progenitor cells. Meanwhile, in the context of metabolism, PKCθ impairs the insulin signaling pathway in skeletal muscle by phosphorylating IRS‐1, thereby promoting the development of type 2 diabetes. Concurrently, at the metabolic level, PKCθ contributes to the pathogenesis of type 2 diabetes by phosphorylating IRS‐1 in skeletal muscle, thereby disrupting the insulin signaling pathway [188]. In addition to these diverse pathological functions, PKCθ holds diagnostic utility: acting as a biomarker, its synergistic interaction with kit IL‐2 tyrosine kinase receptor (KIT) enhances the diagnostic accuracy for gastrointestinal stromal tumors (GIST) [189]. Furthermore, studies indicate that PKCθ paradoxically promotes tumorigenesis, migration, and invasion in triple‐negative breast cancer (TNBC) and enhances human immunodeficiency virus type 1 (HIV‐1) viral replication in acquired immunodeficiency syndrome [190].

### 3.8 PKCζ

PKCζ functions as a central signaling hub, integrating metabolic, proliferative, and immune signals. It plays a pivotal role in regulating cellular adaptive responses under stress conditions [191]. Specifically, PKCζ acts as a signaling nexus within multiple pathways. It participates in the activation of the MAPK cascade, where it serves as a critical mediator in signals governing cell proliferation and differentiation [192, 193]. Furthermore, PKCζ is a key regulator of NF‐κB activation. In this pathway, it phosphorylates the IKK complex, thereby promoting the degradation of IκB [194]. This process releases NF‐κB, enabling its translocation to the nucleus to initiate the transcription of genes involved in inflammation, apoptosis inhibition, and immune responses [195]. Additionally, PKCζ constitutes a major downstream effector of PI3K. Following PI3K‐mediated generation of the lipid second messenger phosphatidylinositol (3,4,5)‐trisphosphate (PIP_3_), PKCζ is recruited to the membrane and phosphorylated at its residue by the PIP_3_‐activated kinase PDK1, leading to its full activation [192, 193, 196].

PKCζ serves as a crucial kinase downstream of the insulin receptor, promoting GLUT4 translocation to the plasma membrane and subsequent glucose uptake. Notably, in hepatocytes, its activation relies on the coordinated action of the microtubule network and the scaffold protein stress‐activated protein kinase‐interacting protein 1 (SIN1) [197]. Functionally, PKCζ primarily regulates metabolism by driving fatty acid β‐oxidation and maintaining glucolipid homeostasis. This role is particularly evident in pathological contexts; for instance, in colon cancer cells stimulated by palmitic acid, PKCζ directly phosphorylates sirtuin 6 (SIRT6) at Thr‐294 [198]. This phosphorylation promotes SIRT6 enrichment on chromatin, thereby activating the transcription of key fatty acid β‐oxidation genes to provide essential energy support for tumor cells [199]. Furthermore, PKCζ plays a significant role in compensatory mechanisms during insulin resistance. Under conditions of hyperglycemia/hyperinsulinemia, PKCζ is activated and drives pancreatic β‐cell proliferation via the mammalian target of rapamycin (mTOR)–cyclin D2 axis, thus helping to preserve insulin secretory capacity [200, 201]. Not only that, under high glucose conditions, PKCζ indirectly affects cell proliferation by enhancing inflammatory factor secretion through the p62‐mediated NF‐κB signaling pathway [202]. Intriguingly, PKCζ can also activate mTORC1 independently of Akt. This alternative activation pathway regulates ribosomal biogenesis and protein translation, thereby influencing critical processes like cell growth [200].

PKCζ plays a significant role in tumorigenesis through its direct regulation of cell proliferation and survival. Specifically, PKCζ suppresses proapoptotic factors, thereby promoting tumor cell survival; conversely, its loss increases caspase‐3‐mediated apoptosis [203]. Furthermore, in pancreatic cancer, PKCζ phosphorylates signal transducer and activator of transcription 3 (STAT3) at Tyr⁷⁰⁵, leading to its activation. This, in turn, drives the expression of downstream proliferative genes (e.g., cyclin D1), ultimately promoting tumor growth and invasion [203]. Notably, PKCζ contributes to cancer progression via unique mechanisms in specific contexts. For instance, the latest research shows that in pancreatic ductal adenocarcinoma (PDAC), PKCζ inhibits the autophagy‐related protein 5 (ATG5)/beclin‐1 pathway by downregulating BCL2‐associated athanogene 3. This action blocks autophagosome formation without causing p62 accumulation—a critical limitation associated with traditional autophagy inhibitors. Importantly, PKCζ knockdown suppresses PDAC cell proliferation and exhibits synergistic antitumor effects when combined with MEK inhibitors [204]. Additionally, PKCζ promotes tumor progression through novel signaling complexes. In TNBC, phosphorylated moesin recruits PKCζ to form a complex. This complex, facilitated by the nuclear protein non‐POU domain‐containing octamer‐binding protein (NONO), translocates to the nucleus. Within the nucleus, PKCζ phosphorylates CREB, activating downstream genes (e.g., alsin rho guanine nucleotide exchange factor, cyclin A1) that drive TNBC progression [205].

In immune‐inflammatory responses, PKCζ and PKCα exhibit bidirectional regulation of NETosis [206, 207]. Specifically, PKCζ activates peptidyl arginine deiminase 4, promoting histone citrullination and thereby driving the release of neutrophil extracellular traps for pathogen capture [207]. In contrast, PKCα inhibits this process. This antagonistic interplay establishes a critical balance, preventing excessive NETosis and subsequent inflammatory tissue damage [208]. Furthermore, PKCζ serves as a key integrator of inflammatory signaling. For example, upon lipopolysaccharide stimulation, the ceramide–PKCζ axis activates NF‐κB [209]. This activation amplifies the production of proinflammatory cytokines, such as TNF‐α and IL‐6, thereby contributing to the pathogenesis of chronic inflammatory diseases [208]. However, PKCζ is not proinflammatory in all cases, for example, in a ras‐induced lung cancer model, PKCζ reduces IL‐6 expression by inhibiting the IL‐6 promoter's histone acetylation of the IL‐6 promoter (acting on the C/EBPβ element) to reduce IL‐6 expression, thereby exerting a tumor‐suppressive effect. PKCζ deficiency instead leads to IL‐6 overexpression and accelerates tumor growth. Expression and accelerated tumor growth [210]. In atherosclerosis models, PKCζ promotes endothelial inflammation by inhibiting the ERK5–kruppel‐like factor 2/endothelial NO synthase pathway, but may also promote endothelial inflammation through other pathways (e.g., modulation of STAT3) involved in anti‐inflammatory homeostasis [211].

Additionally, PKCζ plays a pivotal role in immune cell chemotaxis. PKCζ regulates the macrophage‐stimulating factor (M‐CSF)–Erk pathway at the level of MAPK kinase 1 (MEK1) and carries out regulation of cell proliferation, differentiation, and survival in mononuclear phagocyte cell lines [212]. This mechanism, orchestrated by PKCζ, is also crucial for chemokine‐mediated migration in other contexts. For example, PKC‐ζ regulates stromal cell‐derived factor‐1‐mediated migration and development of human CD34^+^ progenitor cells and plays a central role in the motility and development of hematopoietic stem and progenitor cells. PKCζ rapidly phosphorylates and regulates actin polymerization and cytoskeletal reorganization [213]. Importantly, SDF‐1 chemotactically directed migration of human epidermal stem cells through activation of PKC‐ζ, so inhibition of PKCζ disrupts the formation of epidermal stem cell pseudopods and significantly reduces their migration efficiency [214].

In the realm of neural function, PKCζ and its cleavage product PKMζ exhibit a stage‐specific functional complementarity. Specifically, PKCι/λ (the human orthologue of PKCλ) operates during the early phase of hippocampal LTP, facilitating the initial consolidation of memory by promoting AMPA receptor trafficking to the plasma membrane [215]. Conversely, PKMζ is essential for the sustained maintenance of late‐phase memory storage. Critically, double knockdown experiments confirm that these isoforms act synergistically to ensure memory integrity [216].

### 3.9 PKCι/λ

PKCι/λ exhibits a notable context‐dependent dual functionality within the tumor microenvironment. Notably, it acts as a tumor suppressor implicated in metabolic and immune regulation in cancers such as hepatocellular carcinoma, prostate cancer, and colorectal cancer. Conversely, in basal cell carcinoma and TNBC, PKCι/λ functions as an oncogenic promoter. This functional diversity likely stems from its intricate molecular basis, which is closely associated with factors such as tissue‐specific signaling contexts, divergent tumor microenvironment compositions, and distinct subcellular localization patterns [217, 218].

PKCι/λ orchestrates divergent, tissue‐specific oncogenic programs through distinct molecular mechanisms. In hepatocytes, PKCι/λ deficiency triggers autophagy and enhances oxidative phosphorylation by phosphorylating microtubule‐associated protein 1A/1B light chain 3 at Ser12. This process induces a ROS burst, leading to sustained activation of the transcription factor nuclear factor E2‐related factor 2 (Nrf2), which subsequently drives the expression of proproliferative and antioxidant genes, thereby promoting malignant transformation and tumor development [219]. Differently, within intestinal epithelial cells, PKCι/λ phosphorylates SCAP, promoting its degradation and consequently inhibiting the processing and activation of SREBP2, the master regulator of cholesterol biosynthesis; this pathway contributes to the pathogenesis of aggressive serrated tumors [220, 221]. Conversely, PKCι/λ exhibits potent oncogenic functions in other contexts. In basal cell carcinoma (BCC), phosphorylated PKCι/λ activates glioma‐associated homologue‐1, the core Hedgehog pathway transcription factor, enhancing its DNA‐binding affinity and transcriptional activity, thereby establishing a positive feedback loop that amplifies protumorigenic signaling [222]. Similarly, in TNBC, PKCι/λ mediates the proinvasive effects of inflammatory cytokines (TGF‐β/IL‐1β) via the PI3K–PKCι/λ–NF‐κB axis, facilitating NF‐κB p65 nuclear translocation and subsequent expression of invasion‐promoting genes [223].

Beyond its dual roles in cancer, PKCι/λ serves as a master regulator of cell polarity and embryonic development. Genetic deletion of PKCι/λ in mice leads to severe developmental defects, characterized by a constricted amniotic cavity at embryonic day E7.5, followed by growth arrest and lethality by E8.5. While PKCζ can partially compensate for PKCι/λ function transiently at E7.5, it fails to substitute for its specific roles beyond E9.5, demonstrating the essential and nonredundant requirement for PKCι/λ in late‐stage embryogenesis [224]. Mechanistically, PKCι/λ orchestrates epithelial cell polarity by forming a complex with partitioning‐defective 3 and partitioning‐defective 6 (Par6). Knockout studies reveal that PKCι/λ deficiency disrupts the localization of the tight junction protein zonula occludens‐1 and compromises epithelial architecture. Notably, the initial establishment of polarity foundations can still occur, suggesting its primary role lies in the maintenance rather than the initial assembly of apical‐basal polarity [223]. Within the nervous system, PKCι/λ integrates signaling for synaptic plasticity and neurodegeneration. Upon neuronal activation by glutamate, PKCι/λ is activated via the PI3K–PDK1 axis (phosphorylation at T555). Subsequently, scaffolded by the adaptor protein p62, PKCι/λ phosphorylates the glutamate receptor 1 (GluA1) subunit of AMPA receptors at Serine‐818. This phosphorylation event drives GluA1 trafficking and insertion into the postsynaptic membrane, thereby enhancing synaptic transmission efficacy [225]. Meanwhile, in the context of AD, PKCι/λ exacerbates pathology by phosphorylating sorting‐related receptor with A‐type repeats (SORLA), a protective AD factor. This phosphorylation promotes SORLA's binding to β‐arrestin2 and targets it for lysosomal degradation, consequently accelerating amyloid‐beta (Aβ) deposition. Critically, targeted inhibition of PKCι (e.g., using Auranofin) restores SORLA levels and improves cognitive function in AD models [226].

## 4 The Role of PKC in Disease

**FIGURE 4:**
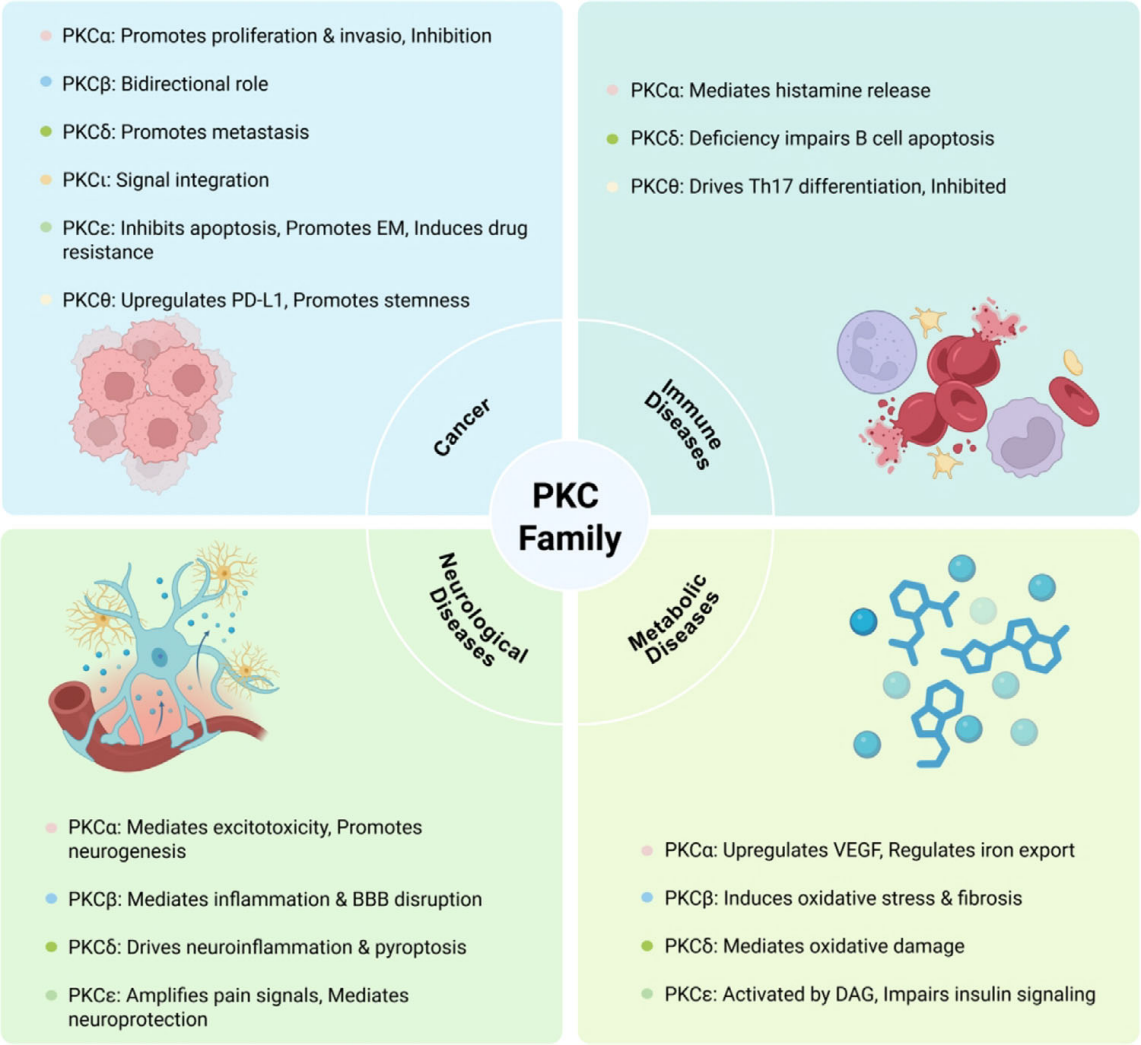
The role of the PKC family in disease. Subtypes (PKCα, PKCβ, PKCδ, PKCι, PKCε, and PKCθ) regulate tumor development, immune system diseases, metabolic diseases, and neurological diseases. (Created with bioRender.com.)

Abnormal activation of the PKC signaling pathway is widely implicated in the pathogenesis of various major human diseases. Therefore, PKC inhibitors have emerged as highly attractive therapeutic agents. The following sections will focus on their therapeutic roles, mechanisms of action, and current research advances in cancers, immune‐related diseases, metabolic disorders, and nervous system diseases (Figure 4). In addition, other disease areas in which the PKC pathway plays a significant role will also be briefly discussed.

### 4.1 The Role of PKC in Tumors

Members of the PKC family (such as PKCα, βII, δ, ε, ι/λ, and θ) play a crucial role in regulating diverse biological processes, including tumor cell proliferation, survival, invasion and metastasis, angiogenesis, and modulation of the tumor immune microenvironment. Aberrant activation of the PKC family is a hallmark of many solid tumors and hematological malignancies, providing a robust rationale for targeting PKC as an anticancer strategy. Notably, the same PKC isozyme may exert markedly different, or even opposing, biological effects depending on the tissue context, tumor type, or stage of disease, which greatly increases the complexity and challenges of developing targeted therapies [227].

Taking PKCα as an example, its biological functions are highly dependent on the tissue environment and tumor type. In colorectal cancer, PKCα activation promotes cytoskeletal remodeling and the degradation of E‐cadherin, thereby enhancing tumor proliferation and invasiveness [228]. In pancreatic cancer, PKCα facilitates cancer cell growth and metastasis by upregulating Raf‐1 [229]. In contrast, in breast cancer, PKCα inhibition paradoxically enhances immune escape mediated by CD^8+^ T cells and promotes metastasis [230], illustrating the complexity and plasticity of its regulatory functions.

PKCβII exhibits dual roles in tumor biology, with context‐dependent expression and function across different cancers. In colorectal cancer, its elevated expression not only accelerates tumor proliferation [231], but is also closely associated with poor prognosis [232]. Conversely, decreased PKCβII expression in other types of cancer correlates with adverse outcomes, suggesting that PKCβII can also function as a tumor suppressor in certain contexts [233].

PKCδ exhibits pronounced tissue specificity and functional diversity. In patients with estrogen receptor‐positive breast cancer, high expression of PKCδ is associated with shorter survival and serves as a tumor‐promoting marker [234]. Additionally, PKCδ promotes liver cancer cell metastasis by activating mitochondrial ROS production and inducing oxidation of HSP60 [235]. However, in different cellular contexts, PKCδ can also exert antitumor effects by regulating the cell cycle and inducing cell death [236]. In human squamous cell carcinoma, PKCδ suppresses the proliferation of cancer stem cells (CSCs) by downregulating p63, thereby inhibiting tumor progression [237]. Its overexpression can also inhibit the malignant phenotype of colon cancer [238]. Notably, inhibition of PKCδ in breast cancer cells leads to upregulation of E‐cadherin, which paradoxically promotes cell migration [239]. In liver cancer, PKCδ knockdown can promote cancer cell survival by inhibiting apoptosis and activating autophagy [240]. These diverse and microenvironment‐dependent functions highlight the complexity of PKCδ as a signaling hub.

PKCι plays a critical role in the integration of signaling pathways across various tumor types. Its downstream signaling varies according to the tumor microenvironment and molecular features. For example, in glioblastoma, it promotes cell proliferation via the PI3K/PKCι/CDK7/CDK2 pathway [241]. In high‐grade serous ovarian cancer, PKCι induces immune suppression through the PKCι/angiomotin/yes‐associated protein 1 signaling pathway [242]. In melanoma, PKCι drives epithelial–mesenchymal transition (EMT) and promotes tumor metastasis via the PKCι–Par6–RhoA signaling cascade [243, 244].

Similar to other PKC isoforms, PKCε demonstrates complex and context‐dependent functional diversity across various tumor types. Although most current research focuses on its protumorigenic effects, PKCε has been shown to promote tumor initiation and progression through multiple signaling pathways in several cancers, including prostate, pancreatic, colorectal, and breast cancer. For example, in prostate cancer, PKCε promotes cell cycle progression via the sustained activation of the ERK signaling pathway, thereby accelerating cell division [245, 246]. In pancreatic cancer, its activation inhibits mitochondrial cytochrome *C* release, thus blocking apoptosis and promoting tumor cell proliferation and survival [247]. Additionally, PKCε phosphorylates migration and invasion inhibitory protein to facilitate cell invasion in colorectal cancer [248], drives EMT and cell migration in breast cancer [249, 250, 251], and regulates VEGF secretion to promote tumor angiogenesis [252]. Notably, PKCε is closely associated with drug resistance, enhancing tumor cell survival by upregulating ABC transporters that increase the efflux of chemotherapeutic agents [253]. While there are numerous reports on its protumor functions, further investigation is needed to elucidate its precise molecular mechanisms and regulatory roles under specific microenvironmental conditions, highlighting both the multidimensional regulatory functions and considerable research value of PKCε in tumor biology.

As a PKC subtype highly enriched in immune cells, PKCθ exhibits pronounced functional heterogeneity. In addition to directly regulating tumor cell proliferation, survival, and migration, PKCθ significantly influences tumor progression by remodeling the tumor immune microenvironment [254]. In TNBC brain metastases and immunotherapy‐resistant metastatic melanoma, PKCθ activates transcriptional programs related to EMT, promotes the generation and maintenance of CSCs with a mesenchymal‐like phenotype, and thereby assists tumor cells in evading immune surveillance [255]. Furthermore, PKCθ enhances glycolysis and upregulates PD‐L1 expression by regulating tumor cell metabolism, which inhibits effector T cell function and leads to a vicious cycle of metabolic‐immune suppression in tumor types such as lung cancer [256]. These findings not only reveal the unique mechanisms of PKCθ in immune and metabolic regulation but also further underscore the dependence of its biological functions on both the tumor microenvironment and cellular context.

Overall, PKC family members in tumor biology demonstrate remarkable heterogeneity, which is further shaped by factors such as the microenvironment, tumor type, and tissue context. At present, the development of highly selective inhibitors targeting the functional diversity of individual PKC isoforms, along with multitargeted and immunotherapy‐based combination strategies, has emerged as a research hotspot. Nonetheless, major challenges—including tumor heterogeneity, limited efficacy of single agents, off‐target toxicity, and contradictory or context‐dependent functions among subtypes—remain to be overcome. Of particular interest, PKC, especially PKCθ, plays a pivotal role in T cell signaling and immune regulation, highlighting its unique potential as a therapeutic target for immune‐related diseases.

### 4.2 The Role of PKC in Immune System Diseases

The PKC family, through the specific distribution and functional regulation of its subtypes in immune cells, serves as a central regulatory hub in autoimmune diseases, inflammatory disorders, allergic reactions, and transplant rejection. Dysregulation of PKC activity directly disrupts immune tolerance, leading to excessive inflammatory responses and tissue damage, thereby highlighting this family as a critical molecular target for the treatment of immune‐mediated diseases.

As a classic PKC subtype, PKCα plays a central role in allergic airway inflammatory diseases. In type I hypersensitivity reactions, PKCα is activated by allergens, which further activates MAPK (ERK), induces histamine release, and triggers allergic responses. Antiallergic peptides can block the phosphorylation of PKC, thereby attenuating allergic reactions [257]. In allergic asthma, eosinophil extracellular traps amplify allergic inflammatory responses and airway hyperresponsiveness via the coiled‐coil domain containing 25–integrin‐linked kinase–PKCα–CREB‐regulated transcription coactivator 1 pathway [258]. In cold‐induced asthma, inhibition of PKCα can alleviate airway inflammation and promote airway remodeling by regulating upstream and downstream signaling pathways [259]. The prominent role of PKCα in allergic diseases highlights its unique clinical value as a central convergence point and regulatory hub for multiple signaling pathways.

In contrast, PKCδ is primarily involved in maintaining the delicate balance between immune tolerance and infection defense. This isoform serves as a key proapoptotic factor in eliminating self‐reactive B cells and also regulates T/NK cell activity, IFN signaling, as well as antifungal immune pathways. In systemic lupus erythematosus (SLE), PKCδ deficiency results in impaired B cell apoptosis, loss of immune tolerance, and subsequent disease progression [260], while indirectly participating in SLE‐associated inflammatory responses through regulation of the IFN/STAT1 pathway [261]. B‐cell depletion therapy (rituximab) and immunosuppressants (such as mycophenolate mofetil) can alleviate clinical symptoms in SLE patients with PKCδ deficiency [260, 261]. In antifungal immune responses, loss of PKCδ induces ferroptosis of myeloid‐derived suppressor cells (MDSCs) via the C‐type lectin receptors–spleen tyrosine kinase (Syk)–CARD9 pathway, thereby compromising antifungal defense and exacerbating tissue damage [262]. The “loss‐of‐function equals pathogenesis” feature of PKCδ highlights its significance as an important molecular marker and potential therapeutic target for regulating autoimmunity, maintaining immune tolerance, and addressing rare immunodeficiency disorders.

PKCθ is predominantly active in T cells and localizes to the immunological synapse, exhibiting high activation levels in autoimmune diseases that are closely correlated with disease activity. In SLE, PKCθ enhances Th17/IL‐17A secretion via germinal center kinase‐like kinase phosphorylation, thereby exacerbating disease activity [263, 264]. In rheumatoid arthritis, aberrantly upregulated PKCθ promotes joint inflammatory responses and Th17 differentiation, contributing to joint destruction and disease progression [263]. In autoimmune neuropathies such as MS, PKCθ serves as a central regulator of Th17‐mediated pathogenic responses, and its targeted inhibition alleviates central inflammation and demyelinating damage [265]. Skin‐expressed PKCθ further drives T cell activation and inflammatory responses, promoting keratinization and abnormal cell differentiation that led to rash formation and chronic inflammation [265]. Notably, selective inhibition of PKCθ can specifically block T cell activation, thereby reducing immune‐mediated rejection during organ transplantation and prolonging graft survival [266]. Given its specific localization in T cells, PKCθ is considered an ideal target for precision immune modulation, as selective inhibition reduces pathological inflammation with minimal impact on basal immune defense [264, 265]. Its inhibitors have demonstrated favorable efficacy and safety in animal models and preclinical studies. Currently, innovative PKCθ‐targeted agents are being evaluated in multiple clinical and preclinical trials, indicating broad translational potential [267, 268, 269].

Overall, the distinct major isoforms of PKC, owing to their precise spatiotemporal distribution and regulatory roles in immune cell signaling, define their pathological and therapeutic relevance across diverse immune diseases. Meanwhile, metabolic disorders such as obesity and diabetes are closely, bidirectionally regulated with immune diseases, in which PKC also serves as a central molecular hub.

### 4.3 The Role of PKC in Metabolic Diseases

The PKC family serves as an indispensable hub in maintaining glucose and lipid metabolic balance and in mediating responses to environmental stress [270]. Each isoform, through specific expression patterns and targeted regulation, orchestrates vascular function, lipid metabolism, insulin signaling, and oxidative stress across key metabolic organs—including the liver, adipose tissue, muscle, and blood vessels—thus forming a complex pathological axis centered on PKCα, PKCβ, PKCδ, and PKCε. This intricate signaling network exhibits extensive potential for multifaceted regulation and precision therapy in the initiation, progression, and outcomes of glucose and lipid metabolism disorders, nonalcoholic fatty liver disease (NAFLD), and diabetic complications.

PKCα serves as a central signaling hub in a variety of metabolic diseases, orchestrating critical processes including glucose and lipid metabolism, inflammatory responses, insulin signaling blockade, and iron homeostasis regulation. In diabetic nephropathy, PKCα activation induces endothelial oxidative stress and upregulates VEGF, leading to proteinuria and glomerular hyperfiltration, while genetic knockout markedly improves renal dysfunction [271]. PKCα also modulates the intestinal macrophage iron exporter Fpn, thereby aggravating iron deposition in diabetes and hemochromatosis, which can be effectively reversed by pharmacological inhibition [272]. Regarding insulin resistance, PKCα impedes GLUT4 translocation via suppression of PI3K/Akt signaling, with tissue‐specific variations in effect [273]. In NAFLD, aberrant activation of PKCα by integrin β1 promotes hepatic lipid droplet accumulation, with knockout or inhibition of PKCα significantly attenuating the fatty liver phenotype [274]. Through its regulation of vascular injury and iron homeostasis across multiple organs, PKCα underscores its irreplaceable value as a therapeutic target.

PKCβ serves as a central regulator of cellular stress and metabolic homeostasis, and is extensively involved in chronic inflammation, oxidative stress, apoptosis, and fibrosis. By modulating vascular stress responses through early growth response protein 1, PKCβ accelerates the development of atherosclerosis and aggravates ischemia–reperfusion injury [275]. In cholesterol homeostasis, PKCβ acts as a negative regulator of the Raf–ERK pathway; its deficiency leads to disrupted cholesterol metabolism, resulting in bile supersaturation and gallstone formation [276]. In diabetic nephropathy, the Syk–PKCβ axis induces oxidative stress and apoptosis, with pathway inhibition effectively alleviating renal injury [277]. In the context of diabetes‐related cardiac injury, PKCβ promotes macrophage polarization and myocardial immune inflammation, contributing to myocardial ischemia–reperfusion injury; targeted inhibition of PKCβ markedly improves myocardial function and reduces infarct size [278]. Furthermore, PKCβ alleviates fibrosis, inflammation, and atrial fibrillation susceptibility via the PKCβ/NF‐κB/TGF‐β pathway [279]. In renal fibrosis and chronic kidney disease, pharmacological inhibition or genetic deletion of PKCβ reverses fibrosis through the PKCβ/TGF‐β/ROS axis and restores oxidative and metabolic balance [280]. This integrated multipathway regulation establishes PKCβ as a key leverage point for the prevention and treatment of metabolic fibrosis.

PKCδ functions as a key mediator of oxidative stress‐induced damage, with its pathological effects demonstrating distinct spatiotemporal dynamics. In nonalcoholic steatohepatitis, PKCδ promotes hepatic oxidative stress and apoptosis [281], contributing to fibrosis, while inhibition of PKCδ via the TGFβ1 pathway significantly attenuates fibrotic progression [282]. At the hypothalamic regulatory level, palmitoylated PKCδ upregulates microglial inflammation through ZDHHC5, indirectly resulting in hepatic lipid metabolism disorders [283]. In renal injury, PKCδ activation of the Cyclic GMP‐AMP synthase–stimulator of IFN genes pathway drives inflammation and interstitial fibrosis [284]. Within vascular aging, PKCδ activation enhances oxidative low‐density lipoprotein (LDL) uptake via NF‐κB [285], exacerbates atherosclerosis, increases ROS levels, and promotes endothelial senescence. The SIRT3/dehydrogenase/reductase 2 axis mitigates oxidative stress and endothelial aging by suppressing PKCδ [286]. In the myocardium, PKCδ and PKCε display dynamic antagonism: excessive PKCδ activation induces mitochondrial damage, whereas appropriate regulation confers antioxidant protection. Such spatiotemporal specificity underscores the need for precise and context‐dependent intervention strategies [287].

PKCε is distinguished by its prominent lipid signal dependence and acts as both a bridge and amplifier in multiple metabolic disorders. In diabetic peripheral neuropathic pain, PKCε amplifies transient receptor potential vanilloid 1 (TRPV1) signaling, thereby increasing pain sensitivity and facilitating neuroinflammatory responses; pharmacological inhibition of PKCε effectively relieves diabetic neuropathic pain and neural injury [288]. In the liver, activation of PKCε by n‐1, 2‐DAG elevates gluconeogenesis and lipogenesis, thereby contributing to the characteristic phenotype of selective insulin resistance [289, 290, 291, 292]. In adipose tissue, PKCε modulates adipocyte secretion and lipolysis, indirectly impacting hepatic insulin signaling and systemic glucose tolerance [290]. Within skeletal muscle, increased plasma membrane sn‐1,2‐DAG specifically activates PKCε (and PKCθ), leading to phosphorylation‐mediated impairment of insulin receptor function; this adverse effect can be partially reversed by exercise intervention [293]. The tissue‐specificity and integrative role of this signaling network make PKCε an ideal therapeutic target for insulin sensitization.

Although PKC subtype‐targeted therapies for metabolic diseases have made notable progress, long‐term safety and tissue selectivity still pose major hurdles. Notably, metabolic disorders are closely linked to neurological diseases, with extensive overlap in molecular mechanisms and signaling pathways—providing a molecular bridge for elucidating metabolism‐related neurological complications.

### 4.4 The Role of PKC in Neurological Diseases

Members of the PKC family profoundly regulate neuronal excitability, synaptic plasticity, and glial inflammation via subtype‐specific spatiotemporal expression in the CNS. Dysregulation of their functions is directly implicated in pain signal amplification, acute brain injury cascades, and neurodegenerative proteinopathies, establishing PKCs as central molecular hubs in the pathological network of neurological diseases. This section focuses on the four key subtypes—PKCα, PKCβ, PKCε, and PKCδ—systematically analyzing their double‐edged roles and targeted intervention strategies in conditions such as sensory abnormalities, stroke, and cognitive impairments.

Among these, PKCα exerts multifaceted and highly dynamic regulatory functions in neurological disorders, encompassing synaptic plasticity, neuronal development, blood–brain barrier (BBB) integrity, and energy metabolism, with pronounced spatiotemporal specificity. In acute cerebral ischemia and stroke, PKCα functions as a central signaling hub, rapidly stimulating glutamate metabolism via the smoothened (SMO)–PKCα–GLT‐1 pathway, leading to neuronal excitotoxicity and cell death. Inhibition of PKCα, or blocking its interaction with GLT‐1, can markedly reduce neural injury and glutamate toxicity [294, 295]. Moreover, PKCα regulates Na^+^/K^+^‐ATPase (NKA)/GluR2/GLT‐1 signaling and BBB function, where excessive activation exacerbates stroke‐related damage, while appropriate modulation provides neuroprotection—highlighting its double‐edged sword nature [296]. In cognitive and neurogenesis disorders, persistent PKCα activation promotes growth factor release, enhances neural stem cell proliferation, neuronal differentiation, and synaptic plasticity, thereby ameliorating learning and spatial memory functions [297]. Conversely, abnormal lipid metabolism results in brain diacylglycerol accumulation and subsequent PKCα activation, mediating impairments in neural stem cell proliferation/differentiation and causing olfactory as well as learning/memory deficits. PKCα inhibition can partially alleviate these neurogenic and cognitive dysfunctions [298]. Thus, the spatiotemporal dynamics and pathological pleiotropy of PKCα establish a theoretical basis for precise interventions.

PKCβ acts as a key integrator of multiple signaling pathways and pathological processes in neurological disorders, exhibiting high cellular and functional specificity. It is critically involved in stroke, inflammation, and metabolic dysfunction. In cerebral ischemia or reperfusion injury, PKCβ activation enhances neuronal Ca^2+^ overload and oxidative stress [299] and, by transducing ERK/NF‐κB signaling, induces proinflammatory cytokine expression, thereby exacerbating neural injury and promoting apoptosis [300]. Inhibition of PKCβ can substantially attenuate such damage [299, 300, 301]. Under intermittent hypoxia, mitochondrial‐localized PKCβ similarly contributes to chronic hypoxic brain injury and pulmonary hypertension by increasing oxidative stress [302]. In hyperglycemic conditions, PKCβ activation elevates vascular endothelial permeability and inflammation, promoting retinopathy, glomerulosclerosis, and neurovascular complications—effects reversible by PKCβ inhibition [303, 304]. In obese mouse models, PKCβ bridges metabolic disturbances with BBB disruption, inflammation, and cognitive dysfunction [305]. In AD, the PKCβII isoform is highly expressed in damaged neurons and plaque regions, while the PKCβI isoform localizes to axons and newly generated neurons; aberrant PKCβ activation promotes neuronal apoptosis and progression of cognitive impairment [306]. By integrating metabolic, inflammatory, and injury‐related pathways through disease‐specific signaling networks, PKCβ represents an ideal target for precision therapeutic development.

PKCδ plays a central and integrative role in the core processes of neurological injury, profoundly influencing the fate of neurons and glial cells by regulating inflammation, pyroptosis, and gene expression, as well as modulating the BBB and the cerebral microenvironment. In cerebral ischemia, AD, and PD, PKCδ promotes neuroinflammation via the NF‐κB signaling pathway, thereby accelerating disease progression [307, 308, 309]. Additionally, PKCδ aggravates brain injury by triggering pyroptosis through NLR Family CARD domain containing 4 (NLRC4) inflammasome aggregation [310]. In cerebrovascular diseases, PKCδ induces abnormal phosphorylation of endothelial barrier proteins, undermining vascular integrity and exacerbating cerebral hemorrhage and cell damage; targeted inhibition of PKCδ can reverse BBB disruption [311, 312]. During epileptic seizures, PKCδ decreases glial glutamate clearance, resulting in neuronal hyperexcitability and worsened excitotoxicity [313]. Serving as a pivotal “bridge” and “amplifier” within the intricate network of neurons, glia, and vasculature, PKCδ, with its broad disease spectrum, multifunctional regulation, and strong therapeutic potential, has emerged as a crucial target for intervention in CNS disorders.

PKCε demonstrates distinct roles in neurological diseases, exhibits high tissue and signaling pathway specificity, and exerts a pronounced “double‐edged sword” function. It plays dual roles in pain sensitization and inflammation, while also effectively mediating metabolic homeostasis and neuroprotection. During pain onset, PKCε activation amplifies sensory neuronal signaling, facilitating chronic pain sensitization. Inhibition at its ATP‐binding site can reverse paclitaxel‐induced chemotherapy neuropathic pain and opioid‐withdrawal mechanical hypersensitivity [314]. Following cerebral ischemia, upregulation of the PKCε–Nampt–Sirt5–ALDH2 axis reduces brain injury, enhances BBB integrity, and mitigates oxidative stress, thus conferring neuroprotective benefits [315, 316]. In PD, PKCε modulates the Trk/PI3K/Akt signaling pathway, supporting dopaminergic neuron repair [317]. PKCε is also pivotal in diabetes‐associated chronic inflammation through the fibroblast growth factor receptor 1/NF‐κB pathway, which correlates with pain severity [318]. In peripheral nerves, PKCε augments TRPV1 function in the carotid body, driving neuroinflammation, allergic responses, and asthma—a pathological reflex loop effectively disrupted by PKCε inhibitors [319]. The unique contributions of PKCε in neurological disorders, characterized by high tissue specificity, signaling hub properties, and bidirectional regulation, highlight its considerable potential for therapeutic development across diverse diseases.

Overall, the distinct subtypes of the PKC family serve as central molecular hubs mediating neuronal excitability, synaptic plasticity, inflammatory responses, and cell survival in neurological diseases, owing to their spatiotemporally specific expression and multidimensional signal regulation. As highly selective modulators and signaling pathway targets continue to be developed, precise intervention in PKC‐related pathological processes is emerging as a key strategy to improve the prognosis of neurological disorders.

## 5 Preclinical and Clinical Research Progress of PKC

As previously described, abnormal activation of PKC subtypes—such as PKCα overexpression in tumors, hyperactivation of the PKCθ signaling pathway in autoimmune diseases, and loss of PKCδ function in neurodegenerative disorders—constitutes core mechanisms driving the progression of various diseases. Targeted correction of these signaling imbalances has become a key therapeutic strategy for such conditions [320]. Currently, drug development targeting these disease‐associated pathways is advancing rapidly, encompassing three main categories: broad‐spectrum inhibitors, subtype‐specific inhibitors, and pathway activators.

### 5.1 Broad‐Spectrum PKC Inhibitors

Taking broad‐spectrum PKC inhibitors as an example, midostaurin (PKC412) is a staurosporine analog derived from Streptomyces species. Owing to its inhibitory activity against PKCα, PKCβ, and PKCγ, as well as multiple receptor tyrosine kinases, midostaurin has demonstrated notable efficacy in clinical trials for solid tumors and acute myeloid leukemia (AML) [321, 322, 323]. In a Phase III clinical trial (NCT00651261) involving AML patients with FMS‐like tyrosine kinase 3 (FLT3) mutations, midostaurin combined with chemotherapy significantly prolonged both overall survival (OS) and event‐free survival [324] and was granted breakthrough therapy designation by the United States Food And Drug Administration (U.S. FDA). In a Phase II study (CPKC412D2201) for systemic mastocytosis (SM), midostaurin achieved a 60% overall response rate, with a median response duration of 24.1 months. Additionally, 78% of patients exhibited marked reductions in bone marrow mast cell infiltration and serum tryptase levels [325, 326]. Despite these therapeutic benefits, most broad‐spectrum inhibitors lack subtype selectivity, often leading to clinical toxicity. As a result, research and development have shifted toward the advancement of subtype‐selective inhibitors in recent years.

### 5.2 Subtype PKC Inhibitors

In the field of subtype‐selective inhibitor development, the PKCβ inhibitor enzastaurin (LY317615), a noncyclic diindolylmaleimide, primarily inhibits PKCβ via ATP‐competitive binding and modulates the PI3K/AKT signaling pathway [327, 328]. However, it failed to improve disease‐free survival in a Phase III clinical trial for diffuse large BCL (DLBCL) (PRELUDE, NCT03263026) [329]. And did not enhance progression‐free survival (PFS) in a Phase II trial for NSCLC (NCT00452413) when combined with chemotherapy [330]. These outcomes are mainly attributed to limited efficacy and inadequate subtype selectivity.

Darovasertib (IDE196) is a first‐in‐class PKCα/β selective inhibitor that received orphan drug designation from the U.S. FDA in May 2022 for the treatment of uveal melanoma (UM) [331]. Approximately 90% of UM patients harbor guanine nucleotide‐binding protein alpha q (GNAQ) or GNA11 mutations, positioning PKC downstream of GNAQ and GNA11 and leading to sustained activation of PKC signaling, thus making PKC inhibition a promising therapeutic strategy [331, 332]. According to Phase I/II clinical data from IDEAYA Biosciences (NCT03947385), darovasertib monotherapy in patients with metastatic UM (mUM) achieved a 1‐year OS rate of 57% (historical control: 37%), with a median OS of 13.2 months [333]. In treatment‐naive mUM patients, combination therapy with the cMET inhibitor crizotinib resulted in an objective response rate (ORR) of 45% and a disease control rate (DCR) of 90%, reaching a DCR of 100% in patients with liver metastases; partial responses were also observed when combined with the MEK inhibitor binimetinib [333]. Phase II/III clinical trials (NCT03947385) are currently underway.

In the field of PKCθ inhibition, sotrastaurin (AEB071) is primarily investigated for the treatment of psoriasis [334] and acute kidney transplant rejection [335]. In a Phase II trial (NCT00403416), sotrastaurin combined with tacrolimus demonstrated efficacy in preventing acute kidney transplant rejection; however, its combination with mycophenolic acid (NCT00492869) was not effective [335, 336]. Another inhibitor, CC‐90005, is a PKCθ allosteric inhibitor that blocks T‐cell activation by suppressing IL‐2 release. It is orally bioavailable and has exhibited notable efficacy in preclinical models of inflammation. A Phase I trial for psoriasis treatment (NCT02443961) has been completed [337].

First‐generation PKCθ inhibitors such as sotrastaurin have shown efficacy in psoriasis; however, their low oral bioavailability (approximately 17%), poor metabolic stability, and significant off‐target effects due to low kinase selectivity have limited their clinical use. To address these issues, macrocyclic PKCθ inhibitors reported in 2024 (e.g., compound 43) have achieved a >100‐fold improvement in kinase selectivity over sotrastaurin by introducing conformational constraints. Moreover, the incorporation of fluorine atoms into the 1,5‐naphthyridine ring lowers basicity, enhancing metabolic stability. These compounds have demonstrated favorable oral bioavailability (58%) in animal models and exhibit strong potential for further development [338]. While PKC inhibition strategies have led to notable advances in the treatment of tumors and immune diseases, activation of PKC signaling pathways in pathological states, such as neuroprotection and metabolic regulation, also represents a promising therapeutic approach.

### 5.3 PKC Activators

In contrast to inhibitor strategies, PKC activators offer a novel therapeutic approach for diseases characterized by signal deficiencies by enhancing the biological activity of specific PKC isoforms. Classic PMA‐like DAG analogues mimic the binding of endogenous DAG to the C1 domain of PKC, thereby broadly activating PKC [339]. In a Phase I trial involving patients with relapsed or refractory hematological malignancies, PMA at doses ranging from 0.063 to 0.125 mg/m^2^ was well tolerated, with main adverse events being transient fever, dyspnea, and phlebitis, and no severe organ toxicity was observed. PMA administration induced phenotypic and gene expression changes associated with malignant cell differentiation, confirming its biological activity; however, its clinical efficacy as a monotherapy was limited [340]. Despite its widespread use in vitro and animal models—as in the induction of airway smooth muscle contraction or modulation of spinal cord NR2B receptor synapse expression [341]—the irreversible activation properties and carcinogenic potential of PMA have hindered its clinical translation.

Bryostatin‐1 is a naturally occurring macrolide isolated from bryozoans that interacts with the C1 domain of PKC, functioning as an activator or inhibitor depending on the context [342]. It primarily activates PKCε and PKCδ (with a particular emphasis on PKCε) to exert neuroprotective effects in various neurological disease models, including AD [343, 344], MS [345, 346, 347], fragile X syndrome (FXS) [348, 349, 350], stroke [351, 352, 353, 354], traumatic brain injury (TBI) [355, 356, 357], and depression [358, 359, 360]. In a Phase II a single‐dose trial (NCT02221947), intravenous administration of 25 µg/m^2^ bryostatin‐1 led to an average increase of 1.83 ± 0.70 points in Mini‐Mental State Examination scores compared with baseline, indicating short‐term cognitive improvement with good tolerability [361]. Another Phase II randomized, double‐blind, placebo‐controlled trial (NRP101 202) validated target engagement through cerebrospinal fluid biomarkers of PKC activation, and further demonstrated that bryostatin‐1 improves cognitive function via PKCε activation [362], with especially notable effects in late‐stage AD patients not receiving memantine and an overall manageable safety profile [363].

In HIV, the eradication of latent viruses within resting CD4^+^ T cells remains a major barrier to achieving a cure [364]. Preclinical studies have demonstrated that bryostatin‐1, acting as a PKC activator (primarily activating PKCα and PKCδ), can induce HIV‐1 LTR transcription [365], significantly activating latent viruses in primary CD4^+^ T cells and astrocytes [366], bryostatin‐1 also exhibits synergistic effects when combined with histone deacetylase inhibitors (such as valproic acid), enhancing the efficiency of latent virus activation and facilitating subsequent immune clearance. Furthermore, it can downregulate the expression of HIV receptors (CD4 and CXCR4) [367, 368], thereby reducing viral cytotoxicity and further supporting its therapeutic potential for HIV. However, a Phase I study (NCT02269605) evaluating single‐dose administration (10–20 µg/m^2^) in HIV‐infected individuals found that bryostatin‐1 was safe and well‐tolerated, but did not effectively activate PKC—potentially due to insufficient plasma concentrations—and no reactivation of latent viruses was observed [369].

The above text provides a detailed discussion of representative PKC inhibitors and activators in both basic research and clinical translation. To facilitate a more intuitive comparison of the research depth, key mechanisms, and current progress status of various compounds under different intervention strategies, the main information on existing PKC inhibitors and activators has been systematically summarized in Tables 2 and 3.

**TABLE 2:** Key advances in preclinical and clinical research on PKC inhibitors.

Type	Drugs	Structure	Target	Combination	Indications	Key result	Mechanism	ID	Phase	References
Broad‐spectrum PKC inhibitors	Midostaurin (PKC412)	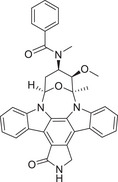	Pan‐PKC	Standard chemotherapy	AML	Combination chemotherapy significantly prolongs survival (approved indication).	Inhibits wild‐type and mutant FLT3, blocks downstream signaling pathways, inhibits leukemia cell proliferation, and induces apoptosis	NCT00651261	III	[324]
			PKCβ	None	SM	Overall response rate of 60%, with a median response duration of 24.1 months	PKCβ is the main target, with consideration of the PI3K/AKT pathway; it inhibits wild‐type and D816V mutant KIT, reducing mast cell proliferation.	CPKC412D2201	II	[325]
	Gö 6983	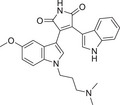	Pan‐PKC	None	Myocardial ischemia/reperfusion injury (I/R)	Significantly improves myocardial contractility in rats with I/R	Inhibition of PKC in PMNs significantly reduces superoxide release and PMN infiltration into the myocardium, thereby improving myocardial contractile function after I/R.	N/A	N/A	[369]
PKC isoform inhibitors	Aprinocarsen (ISIS‐3521, LY900003)	Deoxyribonucleic acid, d(P‐thio) (A‐C‐T‐T‐T‐G‐A‐G‐T‐G‐G‐T‐C‐G‐C‐T‐C‐T‐T‐G)	PKCα	None	Advanced ovarian carcinoma	Limited efficacy, with only one platinum‐resistant patient showing a partial response and no significant objective response	Inhibiting PKCα can block tumor cell proliferation signals and inhibit tumor growth.	N/A	II	[370]
			PKCα	Gemcitabine and cisplatin	Advanced, previously untreated NSCLC	Results not yet released	Inhibiting PKCα can block tumor cell proliferation signals and inhibit tumor growth.	NCT00034268	III	[371]
			PKCα	Carboplatin and paclitaxel	NSCLC	Results not yet released	Specifically inhibits PKC‐α protein expression, blocks tumor cell proliferation, survival, and drug resistance‐related signaling pathways, and can be combined with carboplatin and paclitaxel to synergistically enhance antitumor effects through multiple targets, thereby improving chemotherapy sensitivity.	NCT00017407	III	[372]
	Chelerythrine (CHE)	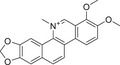	PKCα	PD‐L1	Breast cancer	Tumor growth is significantly inhibited when PKCα inhibitors are used in combination with anti‐PD‐L1 monoclonal antibodies.	PKCα inhibits immune escape in breast cancer by promoting PD‐L1 phosphorylation and degradation, while inhibition of PKCα maintains PD‐L1 stability and accelerates immune escape.	N/A	N/A	[373]
	Darovasertib (IDE196)	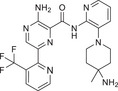	PKCα/β	Binimetinib/crizotinib	Metastatic uveal melanoma (MUM)	Monotherapy: 1‐year OS rate 57% (historical control 37%), median OS 13.2 months 8; combination with crizotinib: ORR 45%, DCR 90%	Inhibiting PKC to block downstream GNAQ or GNA11 mutation signals	NCT03947385	II/III	[331]
	UCN‐01	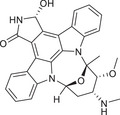	PKCα/β/γ	Cytarabine	Relapsed AML	Low clinical response rate	Blocking S‐phase checkpoints, inhibiting the Akt survival pathway, and activating the JNK proapoptotic signal synergistically reduce AML primitive cells.	N/A	N/A	[374]
			PKCα/β	Prednisone	Cerebral ischemia/hypoxia	No objective response observed	Selective inhibition of PKCα/β, Chk1, and PDK1 promotes tumor cell death, while prednisone alleviates UCN‐01‐related inflammatory responses through immune regulation and enhances targeting of lymphoid tumors.	NCT00045500	I	[375]
			PKCα/β/γ	None	Refractory neoplasms	Single‐agent therapy has limited efficacy, but some patients (especially those with lymphoma) show long‐term disease stability.	Selectively inhibits PKCα/β/γ, blocks cell proliferation and survival signaling pathways, and interferes with the cell cycle	NCT00001444	I	[376]
			PKCα/β/γ	None	Advanced cancer	Results not yet released	Selectively inhibits PKCα/β/γ, blocks cell proliferation and survival signaling pathways, and interferes with the cell cycle	NCT00003289	I	[377]
			PKCα/β/γ	None	Relapsed T‐cell lymphomas	Single‐agent therapy has demonstrated efficacy in certain cases of recurrent T‐cell lymphoma (particularly ALK‐positive ALCL), with a median overall survival of up to 55 months; the overall response rate has not met expectations.	Inhibit PKC, target ALK fusion proteins, and block cell cycle checkpoints	NCT00082017	II	[378]
	Enzastaurin	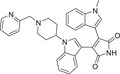	PKCβ	None	DLBCL	No improvement in disease‐free survival was observed.	ATP competitive binding inhibits PKCβ subtypes.	NCT03263026	III(Termination)	[327]
			PKCβ	Erlotinib	NSCLC	Combination chemotherapy did not improve progression‐free survival (PFS).	ATP competitive binding inhibits PKCβ subtypes.	NCT00452413	II	[328]
	Ruboxistaurin (LY333531)	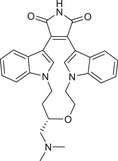	PKCβ	None	Diabetic macular oedema (DME)	Significantly slows the rate of vision loss in patients with long‐term severe DME	Inhibits PKCβ activation, reduces retinal capillary permeability, and decreases neovascularization and vascular endothelial damage	NCT00604383	III	[379]
			PKCβ	None	Recurrent glioblastoma multiforme	Failure to achieve prespecified efficacy endpoints	Inhibiting PKCβ activity, blocking key signaling pathways for tumor cell proliferation and survival, and regulating the immune microenvironment	NCT00297401	III	[380]
	Delcasertib (KAI‐9803)	H‐Cys(1)–Tyr–Gly–Arg–Lys–Lys–Arg–Arg–Gln–Arg–Arg–Arg–OH.H–Cys(1)–Ser–Phe–Asn–Ser–Tyr–Glu–Leu–Ser–Leu–OH	PKCδ	None	Acute anterior ST‐segment elevation myocardial infarction (STEMI)	No significant difference in the primary endpoint	Blocking the binding of PKCδ to the activated C kinase receptor (RACK) reduces myocardial cell necrosis and apoptosis caused by ischemia–reperfusion injury.	NCT00785954	II	[381]
	Rottlerin	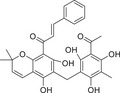	PKCδ	Paclitaxel	Breast cancer	Significantly reduce tumor burden and decrease the formation of pulmonary metastatic nodules	Increases E‐cadherin expression, inhibits Snail 1 and Vimentin expression, inhibits EMT, delays paclitaxel metabolism, and increases its exposure level in plasma	N/A	N/A	[382]
			PKCδ	None	Psoriasis	Significant improvement in psoriasis‐like skin lesions	By arresting the cell cycle at the G0/G1 phase and inhibiting NF‐κB nuclear translocation, PMA induces keratinocytes to release inflammatory factors such as TNF‐α, IL‐6, and IL‐23.	N/A	N/A	[383]
			PKCδ	None	Trimethyltin‐induced neurotoxicity	Reduction in the severity of epileptic seizures	Inhibition of the Nrf2‐dependent glutathione synthesis system and PI3K/Akt antiapoptotic pathway exacerbates oxidative stress and neuronal apoptosis.	N/A	N/A	[384]
	Verproside	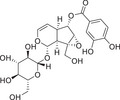	PKCδ	None	Chronic obstructive pulmonary disease (COPD)	Significantly reduces the release of inflammatory factors in lung tissue, excessive mucus secretion, and inflammatory cell infiltration	Inhibits PKCδ activation, reducing inflammatory factors and excessive mucus secretion	N/A	N/A	[385]
	Bryostatin‐1	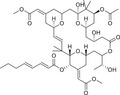	PKCδ	None	Non‐Hodgkin's lymphoma	No objective remission, only 1 case achieved disease stability	Downregulates PKC, promoting tumor cell apoptosis	N/A	II	[386]
			PKCδ	Cisplatin	Advanced or recurrent carcinoma of the cervix	No objective relief and a high disease progression rate	Downregulation of PKC activity inhibits tumor invasion, angiogenesis, cell adhesion, and multidrug resistance‐related pathways	NCT00005965	II	[387]
			PKCδ	Cisplatin	Metastatic or unresectable stomach cancer	Results not yet announced	Downregulates PKC activity, inhibits tumor cell proliferation, invasion, and angiogenesis, and may reverse tumor cell resistance to chemotherapeutic drugs	NCT00006389	II	[388]
			PKCδ	None	Recurrent epithelial ovarian carcinoma	No significant therapeutic effect, no objective remission observed, the main toxicity was myalgia.	Induces tumor cell apoptosis and enhances cytotoxic T‐lymphocyte activity through immune regulation	NCT00004008	II	[389]
			PKCδ	None	Advanced renal cancer	Limited efficacy, with only a few patients achieving disease stability and no objective remission	Inhibiting PKC‐mediated phosphorylation pathways in renal cancer cells reduces the release of proinflammatory factors (such as IL‐6), thereby inhibiting tumor progression.	N/A	II	[390]
			PKCδ	Paclitaxel	Advanced esophageal cancer	It demonstrated some antitumor activity (PR rate 27%), but further development of the regimen was discontinued due to severe myalgia and dose‐dependent toxicity.	Enhances paclitaxel‐induced tumor cell apoptosis through PKC‐mediated signaling pathways	NCT00005599	II	[391]
			PKCδ	Paclitaxel	NSCLC	No significant clinical efficacy was observed; only some patients achieved disease stability, which was accompanied by severe myalgia.	Paclitaxel induces cell cycle arrest, and bryostatin‐1 enhances its‐induced apoptosis by downregulating PKC.	NCT00005849	II	[392]
	Wedelolactone	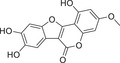	PKCε (indirect)	None	Osteoporosis	Significantly improved osteoporosis in OVX mice	By inhibiting the phosphorylation of the NF‐κB p65 subunit, downstream c‐Fos and NFATc1 activation are blocked, reducing osteoclast‐related genes.	N/A	N/A	[393]
	CP612	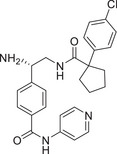	PKCε	None	Hyperalgesia	The short‐term analgesic effect is evident.	Competitively binds to the catalytic domain of PKCε, selectively inhibiting its kinase activity and blocking pain signaling pathways	N/A	N/A	[394]
	PKCZI195.17	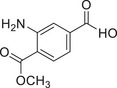	PKCζ	None	Breast cancer metastasis	Significantly reduces the formation of lung metastasis nodules, inhibits tumor growth, and prolongs the survival time of mice, with no obvious toxicity	Inhibiting PKCζ downstream integrin β1 phosphorylation (reducing adhesion) and LIMK/cofilin‐mediated actin polymerization (blocking cytoskeletal rearrangement) thereby blocking tumor cell migration and invasion	N/A	N/A	[395]
	PS432	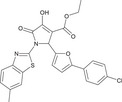	PKCι/ζ	None	NSCLC	The tumor volume in the PS432 group was reduced by approximately 30% compared with the control group.	In vivo, it inhibits PKCι/ζ, downregulates the phosphorylation of CDK7 and CDK2, blocks the cell cycle at the G0/G1 phase, and induces PARP cleavage (an apoptotic marker), thereby reducing tumor cell proliferation.	N/A	N/A	[396]
	Sotrastaurin (AEB071)	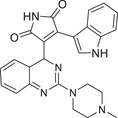	PKCθ/α	tacrolimus	Acute kidney transplant	Achieving good therapeutic results	Selective inhibition of PKCθ/α‐mediated T cell activation	NCT00403416	II	[334]
			PKCθ/α	Mycophenolic acid (MPA)	Acute kidney transplant	High rate of acute rejection, insufficient efficacy when used alone	Selective inhibition of PKCθ/α‐mediated T cell activation	NCT00492869	II	[333]
			PKCθ	Alpelisib	UM	No objective remission was observed, with only a few patients achieving short‐term disease stability.	Simultaneously blocking the PKC/ERK and PI3K/AKT pathways overcomes the compensatory pathway activation caused by single‐target inhibition	NCT02273219	Ib	[397]
			PKCθ	Binimetinib	UM	No objective relief, only a few patients achieved short‐term disease stability.	Inhibiting PKC and MAPK pathways to overcome compensatory pathway activation caused by single‐target inhibition	NCT01801358	Ib	[398]
			PKCθ	None	DLBCL	Significant reduction in tumor volume	Inhibition of PKC downregulates MCT‐1 expression, induces DLBCL cell apoptosis, and G1 phase cycle arrest	N/A	N/A	[399]
	CC‐90005	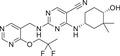	PKCθ	None	Chronic graft‐versus‐host disease (GVHD)	Significantly inhibits swelling of the popliteal lymph nodes (PLN)	Selectively blocking PKCθ‐mediated CBM complex formation reduces the activation of transcription factors such as NF‐κB and inhibits autoimmune responses.	N/A	N/A	[335]
	Aurothiomalate (ATM)	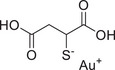	PKCι	None	Lung cancer	Significantly inhibits tumor growth	By combining the PB1 domain of PKCι, blocking its interaction with Par6, inhibiting the downstream Rac1–Pak–Mek1/2–Erk1/2 signaling pathway, thereby inhibiting lung cancer cell proliferation.	N/A	N/A	[400]

**TABLE 3:** Key advances in preclinical and clinical research on PKC activators.

Type	Drugs	Structure	Target	Combination	Indications	Key result	Mechanism	ID	Phase	References
Broad‐spectrum PKC activators	Phorbol 12‐myristate 13‐acetate (PMA)	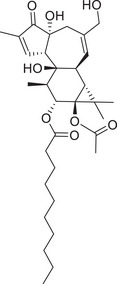	Pan‐PKC	Dexamethasone and choline magnesium trisalicylate (CMT)	Hematologic malignancies	No efficacy or safety data were obtained.	TPA reverses the malignant phenotype of leukemia cells and enhances chemotherapy sensitivity by activating PKC.	NCT01009931	II	[401]
			Pan‐PKC	None	Recurrent/refractory hematological malignancies	Low‐dose TPA shows acceptable safety in patients with relapsed/refractory hematological malignancies and induces leukemia cell differentiation.	Activation of PKC induces the expression of downstream transcription factors MHC‐I and TGF‐β, which are related to differentiation, inhibit tumor cell proliferation, and promote their phenotypic maturation.	N/A	I	[338]
	Ingenol mebutate(PEP005)	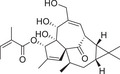	PKCδ	None	HIV	Local application effectively clears AK lesions in HIV‐positive patients and activates latent HIV in the skin via the PKC/NF‐κB pathway without significant systemic immune activation.	Binds to PKCδ, induces its activation and translocation to the cell membrane, activates the downstream NF‐κB signaling pathway, promotes HIV proviral transcription, and breaks the latent state	N/A	N/A	[402]
			Pan‐PKC	CD40 mAb	Breast cancer	The combination group showed a ∼60% reduction in tumor weight compared with the single‐drug group (p < 0.01) and a ∼70% reduction in tumor volume compared with the control group.	Activate the PKC/p38 pathway, reduce the infiltration and inhibitory function of myeloid‐derived suppressor cells (M‐MDSCs), promote their differentiation into antigen‐presenting cells, and increase the proportion of cDC1, thereby reshaping the tumor immune microenvironment	N/A	N/A	[403]
	Prostratin	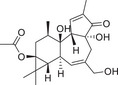	Pan‐PKC	None	Neurogenesis	BrdU+ cell numbers in the ipsilateral and contralateral SVZ increased by ∼40 and ∼30%, respectively, compared with the control group.	Activation of PKC enhances NPC proliferation by synergizing with bFGF signaling without depleting the neural stem cell pool.	N/A	N/A	[404]
	Bryostatin prodrug 8d	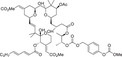	Pan‐PKC	None	HIV	Significantly improves HIV latency reversal efficacy in mouse models	Releases active ingredients to activate the PKC/NF‐κB pathway	N/A	N/A	[405]
PKC isoform activators	Bryostatin‐1	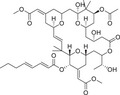	PKCε	None	AD	A single dose improves short‐term cognitive function, and multiple doses restore key functions in patients with severe AD, with good safety.	Activation of PKCε upregulates molecules such as PSD‐95 and BDNF, promoting synaptic repair and neuroprotection.	NCT02221947	IIa	[357]
			PKCε	None	AD	Significantly improves cognitive function (increased SIB scores) with a good safety profile in memantine‐naïve patients with moderate to severe AD	Activation of PKCε promotes synaptic repair and neuroprotection.	NRP101‐202	II	[358]
			PKCε	None	Experimental autoimmune encephalomyelitis (EAE)	Prevents EAE onset	Inhibit the proinflammatory phenotype of DCs and macrophages, reduce proinflammatory cytokine secretion; directly inhibit Th1/Th17 cell differentiation and central nervous system infiltration; promote the formation of an anti‐inflammatory immune microenvironment	N/A	N/A	[342]
			PKCε	Extracellular vesicles (EVs)	EAE	Significantly alleviates neuroinflammation and demyelination in EAE mice	Combines with the PKCε C1 domain to inhibit the NF‐κB pathway and promote M2‐type polarization	N/A	N/A	[341]
			PKCε/α	Exo‐PDGFRα	MS	Significantly promotes oligodendrocyte differentiation and myelin regeneration, while inhibiting neuroinflammation and axonal damage	Activate the PKCε/α pathway, promote oligodendrocyte differentiation, and inhibit neuroinflammation	N/A	N/A	[343]
			PKCε/α	None	Fragile X syndrome (FXS)	Long‐term (13‐week) administration significantly improves autistic phenotype and cognitive deficits in Fmr1 KO2 mice; short‐term treatment is ineffective.	Continuous activation of PKCε/α repairs synaptic plasticity and neural circuit development.	N/A	N/A	[344]
			PKCε/α	None	Stroke	Significantly improved spatial learning and memory in rats	Activates PKCε/α, inhibits the ischemic neuronal apoptosis pathway, reduces cell death in the CA1 region; upregulates BDNF expression, enhances neuronal survival signals; induces dendritic spine formation and maturation, and increases synaptic connection strength.	N/A	N/A	[350]
			PKCε/α	None	Middle cerebral artery occlusion (MCAO)	Significantly reduced mortality and brain swelling with r‐tPA treatment, successfully extending the safe treatment window for tPA to 6 h.	Upregulates PKCε, inhibits PKCα, and downregulates MMP‐9 to alleviate BBB disruption and hemorrhagic transformation after ischemic stroke	N/A	N/A	[347]
			PKCε/α	None	MCAO	Significantly improved survival rates in elderly rats after ischemic stroke, and improved long‐term neurological function and spatial cognition	PKCε‐mediated antiapoptosis, blood–brain barrier protection, and blockade of proinjury pathways following PKCα inhibition	N/A	N/A	[348]
			PKCε/α	None	Cerebral ischemia/hypoxia	Can protect long‐term memory both before and after ischemia	Inhibiting neuronal apoptosis, promoting mushroom‐shaped dendritic spine regeneration, and synaptic vesicle repair	N/A	N/A	[349]
			PKCε/α	None	TBI	Reduces postblast neuroinflammation and secondary brain damage	Downregulation of damaging PKCα, upregulation of protective PKCε, and increased tight junction protein expression effectively repair blast‐induced BBB damage.	N/A	N/A	[351]
			PKCε	None	TBI	Significantly improved inflammatory response, neuronal survival, and behavioral performance	Activate PKCε, inhibit proinflammatory pathways, and promote neuroprotective signals	N/A	N/A	[352]
			PKCε/α	None	Mild traumatic brain injury (mTBI)	Significantly improves cognitive function in mice and repairs synaptic damage	Upregulate ADAM10 and downregulate BACE1 to reduce Aβ production	N/A	N/A	[353]
			PKCε	None	Resistant depression	Significantly improved depressive behavior and associated cognitive deficits	PKCε activation‐mediated BDNF upregulation and enhanced neuroplasticity	N/A	N/A	[354]
			PKCε	None	Depression	High doses can simultaneously enhance spatial memory and alleviate depressive behavior in rats.	Activation of PKC regulates hippocampal synaptic plasticity and neurotrophic/neurotransmitter systems	N/A	N/A	[355]

## 6 Conclusion and Future Directions

Since the discovery of the PKC family, research in this field has witnessed significant progress. Initial investigations were predominantly focused on elucidating the fundamental biochemical properties of PKC. Over time, research has delved deeper into understanding the roles of PKC across diverse physiological and pathological processes. However, translating PKC research from bench to bedside, culminating in the development and approval of novel therapeutics, continues to present substantial challenges.

The first major challenge is the lack of subtype‐specific regulation. PKC isoforms exhibit high functional redundancy and mutual antagonism across different tissues [406]. Due to the minimal spatial topology differences in their catalytic domains when activated, designing rigid structures with high specificity is exceptionally difficult. This inherent structural similarity results in a lack of isoform selectivity for traditional inhibitors. Furthermore, since many kinase inhibitors target the highly conserved ATP‐binding pocket of kinases, achieving selectivity for specific PKC isozymes remains challenging. This limitation leads to significant off‐target effects and associated toxicity. Additionally, strategies using bisubstrate inhibitors have been explored to direct selectivity toward specific PKC isozymes. These inhibitors target both the peptide substrate binding site and the ATP‐binding site. However, bisubstrate inhibitors are typically amphiphilic macromolecules (molecular weight >800 Da), which suffer from poor membrane permeability and consequently low cellular delivery efficiency.

The second major challenge lies in achieving tissue‐selective targeting. The tissue‐specific expression and function of PKC isoforms represent both a critical window of opportunity for precise therapeutic intervention and a significant source of complexity. Individual PKC isoforms cannot be broadly categorized as universally beneficial or detrimental, as they may exert opposing functions in different tissues or at distinct stages of disease progression. Consequently, achieving organ‐specific targeting remains a major research focus.

The third significant challenge involves clinical translation. The intricate nature of PKC‐mediated signaling pathways makes it difficult to understand how to effectively target specific PKC isozymes therapeutically while minimizing off‐target effects. While numerous preclinical studies demonstrate promising results with PKC inhibitors or activators, these findings frequently fail to translate into substantial clinical benefits in human trials. This translation failure is likely attributable to the diversity in experimental models used during preclinical research and the inherent complexity of human pathophysiology.

The inherent challenge of achieving isoform selectivity necessitates the exploration of innovative drug design strategies for PKC modulation. Given the persistent challenge of achieving selectivity, novel drug design strategies warrant exploration. Emerging technologies offer significant promise for enhancing selectivity within the PKC family. Emerging technologies offer promising solutions: proteolysis‐targeting chimeras (PROTACs) represent a transformative strategy by enabling isoform‐selective degradation [407]. This approach exploits the formation of a stable ternary complex (protein of interest–PROTAC–E3 ligase), where target elimination relies on spatial complementarity within the complex rather than solely on catalytic domain binding affinity [408]. This mechanism facilitates the selective degradation of specific isoforms (e.g., PKCδ) despite high catalytic domain homology (>70%) by leveraging conformational selectivity. Further refinement, such as incorporating conformationally constrained linkers (e.g., aromatic rings) to lock isoform‐specific active site conformations, significantly enhances degradation efficiency and specificity, as demonstrated by the conversion of nonselective inhibitors like bryostatin into PKCδ‐selective PROTACs through optimized linker design.

Concurrently, network pharmacology provides a complementary framework through the construction of subtype‐specific PPI networks. By integrating multidimensional data, this approach maps PKC isoform functional divergence, compensatory pathways, and tissue specificity into actionable target landscapes, driving the shift from pan‐PKC inhibition toward precision therapeutics. Future integration of single‐cell sequencing and real‐time phosphoproteomics will further enhance the spatiotemporal resolution of these networks, enabling the systematic dissection of dynamic PKC signaling across cellular contexts [40].

The convergence of additional cutting‐edge technologies—including AI (e.g., leveraging AlphaFold2 to predict activation‐state conformational differences for structure‐guided drug design), advanced in vitro models (organoids, organs‐on‐chips) for improved clinical translatability, and sophisticated delivery systems (surface‐modified lipid/virus‐like particles, tissue‐specific antibody–drug conjugates targeting receptors like CD3)—promises unprecedented insights into PKC regulation in health and disease and addresses the complexities of tissue‐specific targeting.

Collectively, these advances underscore the immense therapeutic potential of the PKC family, a central signaling hub, within a multibillion‐dollar market. The field is decisively transitioning from broad, untargeted inhibition to spatiotemporally precise isoform intervention. The strategic integration of neuro‐immunomodulation, intelligent delivery, network pharmacology, and AI is fundamentally reshaping therapeutic paradigms for cancer, neurological, and immune disorders, heralding a transformative medical era where diseases are defined by molecular mechanisms rather than solely by anatomical location.

In the literature selection process for this review, we acknowledge the presence of potential bias risks. The primary concern is publication bias, where studies with statistically significant positive results are more likely to be published than those with negative or inconclusive findings. This may lead to a systematic overestimation of the functional activity or pathological significance of certain PKC family members when synthesizing conclusions. Additionally, despite efforts to mitigate bias by searching multiple databases and manually tracing references to include relevant studies, we cannot entirely rule out database bias or potential subjectivity during the screening process. Therefore, we advise readers to interpret inferences drawn from the current positive‐result literature with caution. We further advocate for future research to enrich the evidence base through trial registration mechanisms and the publication of negative results, thereby enabling a more comprehensive understanding.

## Author Contributions

Yongqi Li: conceptualization and writing—original draft. Yuhan Jiang: writing—original draft. Zhengxi Hu: data curation. Shenglan Yang: methodology. Longyin Li and Chaohu Xiong: investigation. Ya Gao and Weiguang Sun: writing—review and editing and funding acquisition. Yonghui Zhang: supervision and funding acquisition. All authors have read and approved the final manuscript.

## Ethics Statement

The authors have nothing to report.

## Conflicts of Interest

Author Yonghui Zhang is an Editorial board member of Medcomm. Author Yonghui Zhang was not involved in the journal's review of or decisions related to this manuscript. The other authors declared no conflict of interest.

## Data Availability

The authors have nothing to report.
